# Engagement of distinct epitopes on CD43 induces different co‐stimulatory pathways in human T cells

**DOI:** 10.1111/imm.12642

**Published:** 2016-08-16

**Authors:** Madhura Modak, Otto Majdic, Petra Cejka, Sabrina Jutz, Alexander Puck, Jens G. Gerwien, Peter Steinberger, Gerhard J. Zlabinger, Herbert Strobl, Johannes Stöckl

**Affiliations:** ^1^ Institute of ImmunologyCentre for Pathophysiology, Infectiology and ImmunologyMedical University of ViennaViennaAustria; ^2^ Biopharmaceuticals Research UnitInflammation BiologyNovo Nordisk A/SMåløvDenmark; ^3^ Institute of Pathophysiology and ImmunologyCentre of Molecular MedicineMedical University of GrazGrazAustria

**Keywords:** CD43, co‐stimulation, heterotypic cell adhesion, suppressor T cells, T‐cell polarization

## Abstract

Co‐receptors, being either co‐stimulatory or co‐inhibitory, play a pivotal role in T‐cell immunity. Several studies have indicated that CD43, one of the abundant T‐cell surface glycoproteins, acts not only as a potent co‐receptor but also as a negative regulator for T‐cell activation. Here we demonstrate that co‐stimulation of human peripheral blood (PB) T cells through two distinct CD43 epitopes recognized by monoclonal antibodies (mAb) CD43‐6E5 (T_6E5‐act_) and CD43‐10G7 (T_10G7‐act_) potently induced T‐cell proliferation. However, T‐cell co‐stimulation through two CD43 epitopes differentially regulated activation of nuclear factor of activated T cells (NFAT) and nuclear factor‐*κ*B (NF‐*κ*B) transcription factors, T‐cell cytokine production and effector function. T_6E5‐act_ produced high levels of interleukin‐22 (IL‐22) and interferon‐*γ* (IFN‐*γ*) similar to T cells activated via CD28 (T_CD__28‐act_), whereas T_10G7‐act_ produced low levels of inflammatory cytokines but higher levels of regulatory cytokines transforming growth factor‐*β* (TGF‐*β*) and interleukin‐35 (IL‐35). Compared with T_6E5‐act_ or to T_CD__28‐act_, T_10G7‐act_ performed poorly in response to re‐stimulation and further acquired a T‐cell suppressive function. T_10G7‐act_ did not directly inhibit proliferation of responder T cells, but formed stable heterotypic clusters with dendritic cells (DC) via CD2 to constrain activation of responder T cells. Together, our data demonstrate that CD43 is a unique and polarizing regulator of T‐cell function.

**Abbreviations**
APC: antigen‐presenting cells
AP‐1: activator protein 1
CB: cord blood
DC: dendritic cells
IFN‐*γ*: interferon‐*γ*
IL: interleukin
mAb: monoclonal antibody
MLR: mixed leucocyte reaction
NFAT: nuclear factor of activated T cells
NF‐*κ*B: nuclear factor‐*κ*B
PB: peripheral blood
PBMC: peripheral blood mononuclear cells
T_10G7‐act_: peripheral blood T cells activated via CD3/CD43‐10G7
T_6E5‐act_: peripheral blood T cells activated via CD3/CD43‐6E5
T_CD28‐act_: peripheral blood T cells activated via CD3/CD28
TCR: T cell receptor
TGF‐*β*: transforming growth factor‐*β*

## Introduction

CD43 (sialophorin, leukosialin) is a conserved, transmembrane sialoglycoprotein expressed on most haematopoietic cells except resting B cells and erythrocytes.^1^ It extends ≈ 45 nm from the cell surface and is one of the most abundant molecules expressed on leucocytes.^2^, ^3^ Several studies have addressed the function of CD43 during the last 30 years, but its physiological role is still unclear and is particularly controversial in T cells.

In human and murine T cells, CD43 has been shown to synergize with T‐cell receptor (TCR) signalling to induce T‐cell activation and proliferation independent of CD28 co‐stimulation.^4^, ^5^ Signal transduction through CD43 induces Ca^2+^ mobilization. When cross‐linked with monoclonal antibodies (mAbs), T‐cell stimulation via CD43 leads to activation of the mitogen‐activated protein kinase pathway and further induces the DNA binding activity of nuclear factor‐*κ*B (NF‐*κ*B), nuclear factor of activated T cells (NFAT) and activator protein 1 (AP‐1) transcription factors.^6^, ^7^, ^8^ Downstream of T‐cell co‐stimulation, CD43 triggers various target genes that may exhibit some overlap with CD28 co‐stimulation.^9^ T‐cell co‐stimulation via CD43 in the presence of TCR signalling has been shown to not only promote interferon‐*γ* (IFN‐*γ*) production by CD4^+^ as well as CD8^+^ T cells but also to negatively regulate T helper type 2 differentiation.^10^, ^11^, ^12^

Contrary to the co‐stimulatory role of CD43 reported in these studies, CD43 has been suggested to negatively regulate T‐cell activation.^13^ T cells from CD43‐deficient mice are hyper‐responsive to various mitogenic stimuli *in vitro* as well as *in vivo*.^13^, ^14^ Physical properties, such as large size and negatively charged surface, that in turn create a steric barrier for cell–cell contact, are mainly thought to be responsible for negative regulation of T‐cell adhesion, T‐cell–antigen‐presenting cell (APC) interaction and therefore of T‐cell activation by CD43. Likewise, TCR signalling has been reported to induce selective exclusion of CD43 from the immunological synapse to a distal polar complex.^15^ On the other hand, CD43 along with MHC‐I molecule is involved in spontaneous T‐cell conjugate formation, an initial step in T‐cell activation.^16^ Furthermore, expression of only cytoplasmic domain of CD43 in CD43^−/−^ T cells could reverse the hyper‐proliferative effect of CD43 deficiency. The ectodomain of CD43 did not seem to interfere with the T‐cell–APC interaction.^17^ These observations suggest that negative regulation of T‐cell activation via CD43 is mainly facilitated by an intracellular mechanism and is not merely a phenomenon of a physical barrier function. Additionally, CD43^−/−^ mice showed increased numbers of antigen‐specific CD8^+^ T cells compared with wild‐type mice during the course of viral response after the initial peak of expansion, indicating an important role of CD43 during the contraction of an immune response.^18^ Hence, CD43 seems capable of acting as both a positive and a negative regulator of T‐cell responses.

To elucidate the co‐stimulatory role of CD43 in T‐cell activation, we took advantage of two well‐defined CD43 mAbs 6E5 and 10G7 that bind to different, non‐overlapping epitopes on human CD43.^19^, ^20^ More importantly, previous studies have demonstrated that targeting CD43 with these two mAbs has different functional effects on T‐cell conjugate formation with APC.^16^ We demonstrate in this study that engagement of CD43 on human peripheral blood (PB) T cells via two distinct epitopes induces proliferation of T cells, which occurs in large cellular clusters. Yet, targeting of the two epitopes on T cells exerts polarizing effects such as differential activation of transcription factors, cytokine production and also effector functions. T cells co‐stimulated via the CD43‐6E5‐defined epitope produced high levels of IFN‐*γ* and interleukin‐22 (IL‐22) similar to CD28 co‐stimulation, but only low amounts of IL‐4 and IL‐17. In contrast, stimulation of PB T cells with mAb CD43‐10G7 resulted in poor production of all analysed cytokines except for inhibitory cytokines transforming growth factor‐*β* (TGF‐*β*) and IL‐35. Indeed, T_10G7‐act_ showed a suppressive function, which was not critically dependent on these soluble factors but was mediated by inhibiting the T‐cell stimulatory function of APC. The suppressive T_10G7‐act_ cells formed stable heterotypic clusters with co‐cultured dendritic cells (DC) primarily via CD2/CD58, to further hinder the activation of responder T cells by DC. Taken together, our data demonstrate that CD43 is a unique co‐receptor that can exert differential polarization of T‐cell function, through its different epitopes.

## Materials and methods

#### Media, reagents and chemicals

Cells were cultured in RPMI‐1640, supplemented with 2 mm l‐glutamine, (both Gibco Ltd., Paisley, UK), 100 U/ml penicillin, 100 μg/ml streptomycin (PAA Laboratories, Pasching, Austria) and 10% fetal calf serum (Gibco). Ionomycin and PMA were purchased from Sigma‐Aldrich (St Louis, MO). Recombinant human granulocyte–macrophage colony‐stimulating factor and IL‐4 were kindly provided by Novo Nordisk A/S (Bagsværd, Denmark). IL‐2 was purchased from Peprotech (Rocky Hill, NJ).

#### Antibodies

The following murine mAbs were generated in our laboratory: negative control mAb VIAP (against calf intestine alkaline phosphatase), 6B7 (CD11a), 6E5 and 10G7 (CD43), 3G10 [CD25‐phycoerythrin (PE)], VIP1 (CD71‐Biot), VIM3 (CD97), L243 (HLA‐DR). The mAb MEM‐93 (CD45 RA‐Biot) was a kind gift from Václav Hořejší (Prague, Czech Republic). Hybridomas producing mAb TS2/18 (CD2), mAb TS.1/18 (CD18), mAb W6/32 (MHC class I) and G28‐5 (CD40) were obtained from the American Tissue Culture Collection (ATCC; Manassas, VA). The following mAbs were purchased: mAb FN50 (CD69‐FITC) (BD Biosciences, San Jose, CA); mAb 10F3 (CD28) and mAb against human IL‐4 (MP4‐25d2‐PE) (Invitrogen Carlsbad, CA); mAb HB15a (CD83‐PE) (Immunotech, Marseille, France); mAb B7‐2 (CD86‐PE) (Caltag Laboratories, Buckingham, UK); mAb EH12.1 (PD‐1‐PE) (BD Pharmingen, San Diego, CA); mAb L3D10 (CTLA‐4‐PE) (Biolegend, San Diego, CA); mAbs against human IL‐27/IL‐35 EBI3 subunit (607201‐AlexaFluor 488); human IFN‐*γ* (25723‐PerCP); human IL‐22 (142928‐allophycocyanin) (R&D Systems Inc. Minneapolis, MN) and FOXP3 (259D/C7‐AF647) (BD Biosciences, San Jose, CA). OKT3 (CD3) was obtained from Jansen‐Cilag (Vienna, Austria).

#### Isolation of primary T cells and generation of monocyte‐derived DC

Buffy coats from healthy donors were purchased from either Austrian Red Cross or University Clinic for Blood Group Serology and Transfusion Medicine, Medical University of Vienna (both, Vienna, Austria). To isolate peripheral blood mononuclear cells (PBMC), heparinized buffy coats were further separated by standard density gradient centrifugation (450 ***g*** for 30 min at room temperature) with Ficoll‐Paque^™^ Plus (GE Healthcare, Chalfont St Giles, UK). Subsequently, total T (CD3^+^) cells were obtained via depletion of CD11b^+^, CD14^+^, CD16^+^, CD19^+^, CD33^+^ and MHC class II^+^ cells from total PBMC. CD4^+^ and CD8^+^ T cells were also obtained by negative selection and monocytes were separated by positive selection using the MACS technique (Miltenyi Biotec, Bergisch Gladbach, Germany) as described previously.^21^ For isolation of CD4^+^ CD25^+^ regulatory T cells, CD4^+^ T cells were further incubated with CD25 antibody and were separated by positive selection using MACS. Naive T cells were isolated from umbilical cord blood (CB). CB samples from healthy donors were collected during full‐term deliveries. Ethical approval was obtained from the Medical University of Vienna, institutional review board. Informed consent was provided in accordance with the Declaration of Helsinki. Briefly, T cells were isolated from CD34‐depleted mononuclear cells obtained from CB, using the same protocol as described above. Purity of total T cells (PB T plus CB T cells), CD4^+^ and CD8^+^ T cells was checked routinely. Purity of each cell population was found to be ≥ 97%. Monocyte‐derived DC were generated by culturing purified monocytes for 7 days with a combination of granulocyte–macrophage colony‐stimulating factor (50 ng/ml) and IL‐4 (35 ng/ml).^21^

#### T‐cell proliferation assay

MAXISORP Nunc‐Immuno plates (Thermo Scientific, Waltham, MA) were coated overnight at 4° with either CD3 mAb (OKT3) alone or in combination with CD28 mAb (10F3) or one of the CD43 mAbs (6E5 or 10G7). All mAbs were used at 5 μg/ml. The plates were then washed to remove unbound mAbs and purified T cells (2 × 10^5^/well) were added to the respective wells. T‐cell proliferation was monitored, measuring [methyl‐^3^H]thymidine (PerkinElmer, Inc. Waltham, MA) incorporation at day 3. Cells were harvested 18 hr after adding [methyl‐^3^H]thymidine (0·05 mCi/well) and incorporated thymidine was detected on a microplate scintillation counter (Topcount; Packard, Meriden, CT) as counts per minute. Assays were performed in triplicates.

#### Mixed leucocyte reaction

For mixed leucocyte reaction (MLR) purified T cells (2 × 10^5^ cells/well) were stimulated with allogeneic DC (5 × 10^4^ cells/well). Experiments were performed in 96‐well round‐bottom cell culture plates in the presence of RPMI‐1640 medium (Mock) or indicated cell supernatants, as described previously.^22^ T‐cell proliferation was monitored, measuring [methyl‐^3^H]thymidine incorporation at day 5. Assays were performed in triplicates.

#### Flow cytometry analysis

For membrane staining, cells (2 × 10^5^) were incubated with either unconjugated or conjugated mAbs for 30 min at 4°. For unconjugated mAbs, Oregon Green^®^ 488‐conjugated goat anti‐mouse IgG antibody (Life Technologies, Carlsbad, CA) and for biotinylated mAbs, PE‐conjugated streptavidin was used as the second‐step reagents.

Intracellular cytokine production was determined by pre‐treating the activated PB T cells, for 12 hr with 5 μm monensin (Sigma‐Aldrich) and then by fixing cells in FIX‐solution for 20 min at room temperature before incubating with the respective mAbs along with PERM‐Solution (both, AN DER GRUB Bio Research GmbH, Kaumberg, Austria) for 20 min at room temperature. Flow cytometry analyses were performed using FACScalibur (Becton Dickinson, Franklin Lakes, NJ).

Before FOXP3 staining, cell surface antigens (CD45RA) were stained as described above. Foxp3/Transcription factor staining buffer set (eBioscience Inc., San Diego, CA) was used for intracellular FOXP3 staining. Briefly, The cells were fixed with fixation buffer in the dark at room temperature for 20 min. Cells were then incubated with AF647 anti‐FOXP3 mAb or isotype control mAb in permeabilization buffer in the dark at room temperature for 30 min. Flow cytometry analyses were performed using LSRFortessa (Becton Dickinson).

#### Analysis of duration of CD43 mAb binding

Peripheral blood T cells were incubated with biotinylated CD43‐6E5 or CD43‐10G7 mAb at 4° for 1 hr. An initial binding of CD43 mAbs at 0 hr was immediately analysed by flow cytometry. Part of the labelled cells were maintained at 4°. For analysis at 37°, the labelled T cells were incubated with plate‐bound CD3 mAb, to ensure survival of T cells throughout 3 days of culture. At the indicated time‐point, cells were labelled with PE‐conjugated streptavidin as a second‐step reagent and were analysed by flow cytometry.

#### Determination of cytokine production

Peripheral blood T cells were activated via CD3/CD28 (T_CD28‐act_) CD3/CD43‐6E5 (T_6E5‐act_) or via CD3/CD43‐10G7 (T_10G7‐act_) as described above. At day 3 supernatants were harvested and were pooled from triplicate wells. Supernatants were then used for measuring T‐cell cytokines. Cytokines including IL‐2, IFN‐*γ*, IL‐4, IL‐13, IL‐17, IL‐22, IL‐10, TGF‐*β* were measured using the Luminex100 System (R&D Systems Inc.) as described in the manufacturer's protocol. All measurements were performed in duplicates.

#### Re‐stimulation of T cells

T_CD28‐act_, T_6E5‐act_ and T_10G7‐act_ were harvested at day 3 and were then further cultured for another 4 days in fresh media without stimulation. T cells were then re‐stimulated (2 × 10^5^ cells/well) via plate‐bound CD3/CD28, CD3/CD43‐6E5 or CD3/CD43‐10G7 in the presence or absence of exogenous IL‐2 (20 U/ml). T‐cell proliferation was analysed at day 3 by [methyl‐^3^H]thymidine incorporation. Assays were performed in triplicates.

#### T‐cell suppression assay

T_6E5‐act_, T_10G7‐act_ and T_CD28‐act_ were harvested at day 3 and rested in fresh medium without any stimulus for 4 days, as described above. T cells were then either irradiated (30 Gy, 137Cs source) or pre‐treated with 1% formaldehyde. Pre‐activated T cells, at various cell numbers, were then co‐cultured either with responder PB T cells (1 × 10^5^ cells/well) stimulated with immobilized CD3/CD28 mAb or in an allogeneic MLR with DC (5 × 10^4^ cells/well) and responder PB T cells (1 × 10^5^ cells/well). Where indicated, freshly isolated regulatory T cells were added to allogeneic MLR. Per cent suppression was calculated as described previously.^23^ For T cells activated via plate bound mAbs, proliferation was measured at day 3 and for an MLR at day 5 by [methyl‐^3^H]thymidine incorporation.

#### Real‐time PCR

Total cellular RNA was isolated using peqGOLD TriFast (Peqlab, Erlangen, Germany) with chloroform extraction, followed by isopropanol precipitation according to the manufacturer's protocol. The cDNA was generated using the Revert Aid MuLV‐RT kit (Fermentas, Burlington, Canada) using Oligo‐dT(18 mer) primers according to the manufacturer's protocol and was stored at −20° until further use. Quantitative real‐time PCR was performed with a CFX96 Real‐Time PCR Detection System (Bio‐Rad, Hercules, CA) using SYBR Green qPCR master mix (Quanta Biosciences, Gaithersburg, MD) for detection. *CD3E* was used as an endogenous reference gene.^24^ Specific primers for human *IFNG*,* IL4*,* IL22*,* EBI3*,* p35*,* FOXP3*,* CD3E and p28* were designed using the software primer 3 plus 25 and were synthesized at Sigma‐Aldrich (see Supplementary material, Table S1). Data analysis was performed using CFX manager software (Bio‐Rad).

#### Cell aggregation assay

Dendritic cells were labelled with 1 μg/ml CellTrace^™^ Oregon Green^®^ 488 (carboxy‐DFFDA‐SE) (Invitrogen) in PBS per 1 × 10^7^ cells, as per the manufacturer's protocol. The DC were then resuspended in complete RPMI medium. Labelled DC (5 × 10^4^ cells/well) were added to irradiated pre‐activated T cells (1 × 10^5^ cells/well). Where indicated, T_6E5‐act_, T_10G7‐act_ or T_CD28‐act_ cells were pre‐treated with either blocking anti‐LFA‐1 (CD11a/CD18) or CD2 mAbs (10 μg/ml), before irradiation. Images were taken using Nikon DS‐Fi1c under an inverted Nikon Eclipse Ti‐S fluorescence microscope (Tokyo, Japan) with 10 × magnification. Images were captured at three different locations in each well per assay.

Association of five or more cells (DC and T cells) was designated as a heterotypic cluster. The area of a heterotypic cluster was calculated using the image analysis program fiji.^26^ Briefly, the boundary of an individual heterotypic cluster was defined manually. The scale of an image was calibrated according to the scale bar and the area of each heterotypic cluster was then analysed using fiji.^26^ Number of DC per heterotypic cluster was determined by using definiens analysis software (Definiens AG, Munich, Germany). Oregon Green^®^ 488‐positive DC were detected by stain intensity and were further segmented on the basis of preset thresholds for morphology and area of an individual cell.

For analysis of DC–T‐cell clustering by flow cytometry, DC were labelled with CellTrace^™^ Oregon Green^®^ 488 as described above and pre‐activated T cells (either T_6E5‐act_, T_10G7‐act_ or T_CD28‐act_) were labelled with 5 μm CellTracker^™^ Red CMTPX dye (Invitrogen) in serum‐free medium per 1 × 10^7^ cells, as per the manufacturer's protocol. Both DC and pre‐activated T cells were then resuspended in complete RPMI medium. Where indicated, pre‐activated T cells were pre‐treated with respective blocking mAbs. DC (5 × 10^4^ cells/well) were added to irradiated pre‐activated T cells (1 × 10^5^ cells/well). The cells were analysed by flow cytometry after 30 hr. Double‐positive cells were counted as T cells and DC clusters.

#### Multi‐channel reporter cell line assay

To analyse the activation of downstream signalling pathways, a multi‐channel reporter cell line Jurkat E6 expressing reporter gene under the control of NF‐*κ*B, NFAT and AP‐1 promoter element was used as described previously.^27^ Briefly, a reporter cell line was generated by introducing constructs encoding NF‐*κ*B‐CFP, NFAT‐eGFP and AP‐1‐mCherry into Jurkat E6 cells. A cell clone that was negative for fluorescent proteins in an unstimulated state and strongly up‐regulated CFP, eGFP and mCherry expression upon PMA/ionomycin treatment was selected for further use.^28^ The reporter cells were activated via plate‐bound CD28, CD43‐6E5 or CD43‐10G7 mAbs along with CD3 mAb. To assess the activation of the respective transcription factors, cells were harvested after 12 hr and expression of eGFP, CFP and mCherry were measured by flow cytometry using LSRFortessa (Becton Dickinson).

#### Statistical analysis

Statistical analysis was performed using graphpad prism software (GraphPad, La Jolla, CA). Unpaired, two‐tailed Student's *t*‐test followed by Holm–Šídák test for multiple comparisons was performed and *P*‐values < 0·05 were considered significant. Significant values are represented as *P < 0·05, **P < 0·01, ***P < 0·001.

## Results

### CD43 mAb 6E5 but not CD43‐10G7 down‐regulates cell surface expression of CD43

**Figure 1:**
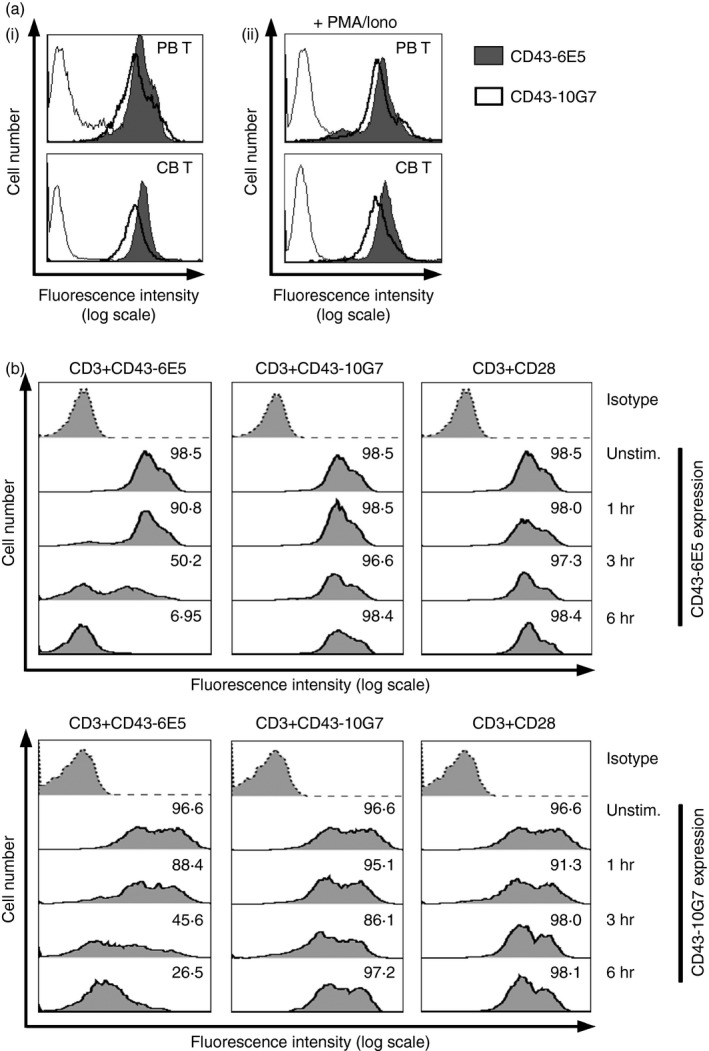
Cross‐linking of monoclonal antibody (mAb) CD43‐6E5 but not CD43‐10G7 down‐regulates CD43 expression. (a) Expression of CD43 epitopes defined by CD43‐6E5 (grey‐filled histograms), CD43‐10G7 (thick open histograms) compared with isotype control (thin open histograms) on unstimulated (i) and PMA/ionomycin (100 nm for 12 hr) stimulated (ii) peripheral blood (PB) T and cord blood (CB) T cells. Gate was set on live cell population (not shown). Data are representative of two independent experiments with four different donors for each PB T and CB T cells. (b) Expression profile of CD43‐6E5 and CD43‐10G7 (thick filled histograms) on unstimulated T cells, T cells stimulated via plate‐bound CD3/CD43‐6E5, CD3/CD43‐10G7 and CD3/CD28 for indicated time‐points. Reactivity of isotype control is also shown (dotted filled histograms). Numbers indicate percentage of positive cells. Gate was set on live cell population (not shown). Data shown are representative of three independent experiments with three different donors.

Previous studies have shown that both CD43 mAbs bind to different, non‐overlapping epitopes on human CD43.^19^ As a result of *O*‐linked glycosylation CD43 exists in two isoforms of size 115 000 and 135 000 MW.^29^ The 115 000 MW isoform is expressed on naive cells, whereas the 135 000 MW isoform of CD43 is associated with T‐cell activation.^30^ Results presented in Fig. 1(a(i)) demonstrate that both CD43 mAbs react with unstimulated PB T cells and CB T cells with similar intensity. Both CD43 mAbs also bound efficiently to PB T as well as CB T cells upon stimulation with PMA/ionomycin (Fig. 1a(ii)). Furthermore, PB T cells stimulated with PMA/ionomycin uniformly expressed the two defined epitopes, similar to unstimulated PB T cells (see Supplementary material, Fig. S1a). The 135 000 MW isoform of CD43 is constitutively expressed more on resting CD8^+^ T cells than CD4^+^ T cells.^31^ CD43‐6E5 and CD43‐10G7 mAbs showed comparable reactivity to PB CD4^+^ and CD8^+^ T cells (see Supplementary material, Fig. S1b). Binding studies showed that two mAbs react with CD43 on PB T cells with similar affinity (see Supplementary material, Fig. S1c). The two CD43 mAbs also showed similar duration of binding as analysed over the period of 3 days (see Supplementary material, Fig. S1d). Additionally, both CD43 mAbs showed reactivity with Bw5417 cells retrovirally transduced to express human CD43 (Bw‐CD43);^32^ but not with the parental Bw5417 cells (Bw) (see Supplementary material, Fig. S1e). Bw5417 cell line lacks C2GnT glycosyltransferase that initiates core 2 *O*‐glycan branching and can express only 115 000 MW isoform of CD43.^33^ Moreover, two CD43 mAbs bind to parental CD43^+^ CEM lymphoid T‐cell line but not to a CD43‐deficient CEM cell line.^20^ Together data suggest that CD43 mAbs 6E5 and 10G7 are specific for human CD43 and bind to different epitopes present on both isoforms of CD43.

Crosslinking of CD43 with mAbs induces T‐cell co‐stimulation and also modulates the cell surface expression of CD43 on leucocytes.^4^, ^6^, ^7^, ^8^, ^9^, ^10^, ^34^ Therefore, we next analysed whether cell surface expression of CD43 is modulated by our CD43 mAbs. Results presented in Fig. 1(b) demonstrate that CD43 is strongly down‐regulated from cell surface upon activation with plate‐bound CD3/CD43‐6E5 but not with CD3/CD43‐10G7 or CD3/CD28. Down‐modulation of CD43 surface expression by CD43‐6E5 was fast and efficient and T cells were almost CD43‐negative after 6 hr of stimulation with CD3/CD43‐6E5 (Fig. 1b). Down‐regulation of CD43 surface expression by CD43‐6E5 could only be observed in the presence of TCR signalling. T‐cell stimulation with plate‐bound CD43‐6E5 alone did not modulate CD43 expression on T cells (see Supplementary material, Fig. S2). CD43 expression was restored to basic levels after 3 days of culture (data not shown).

Hence, both CD43 mAbs CD43‐6E5 and CD43‐10G7 show similar affinity and comparable expression profile on various T‐cell subsets, but differ in their ability to modulate CD43 cell surface expression (Fig. 1, and see Supplementary material, Fig. S1).

### Co‐stimulation upon engagement of CD43 with mAb 6E5 or 10G7 induces T‐cell proliferation

**Figure 2:**
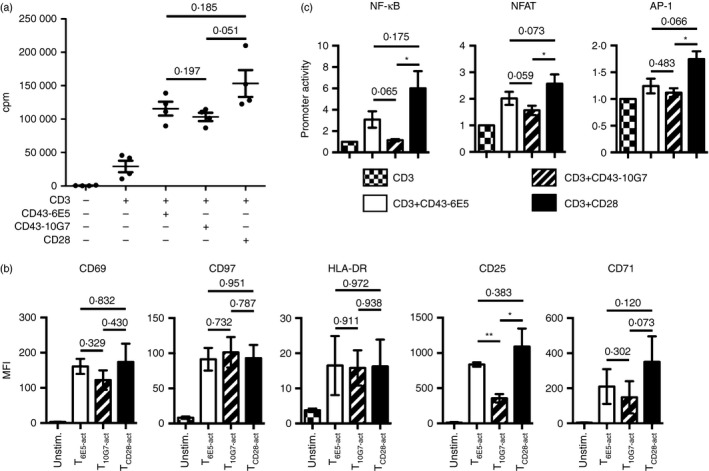
Co‐stimulation via CD43 monoclonal anitbodies (mAbs) induces peripheral blood (PB) T‐cell activation and proliferation. (a) PB T cells were activated via plate‐bound mAbs, CD3, CD3/CD43‐6E5, CD3/CD43‐10G7 or CD3/CD28. Proliferation was measured by analysing [methyl‐^3^H]thymidine incorporation (no. of experiment = 4, no. of donors = 4). (b) PB T cells were activated with the respective plate‐bound mAbs and analysed after 48 hr for expression of various cell surface markers by flow cytometry. Gate was set on live cell population (not shown). Graphs show mean fluorescence intensities of CD69, CD97, HLA‐DR, CD25 and CD71 on unstimulated T cells (checkered bars), T_6E5‐act_ (open bars), T_10G7‐act_ (striped bars) and T_CD__28‐act_ (black bars) (no. of experiments = 3, no. of donors = 3). (c) Activation of nuclear factor‐*κ*B (NF‐*κ*B), nuclear factor of activated T cells (NFAT) and activator protein 1 (AP‐1) transcription factors via CD43. Jurkat E6 reporter cells were activated via indicated plate‐bound mAbs for 12 hr and were analysed for the expression of reporter genes by flow cytometry. Graphs show mean promoter activity values of NF‐*κ*B (CFP), NFAT (eGFP) and AP‐1 (mCherry) of Jurkat E6 reporter cells, activated via plate‐bound CD3/CD43‐6E5, CD3/CD43‐10G7 and CD3/CD28, normalized to promoter activity of Jurkat E6 cells activated via immobilized CD3 alone (no. of experiments = 3). (a–c) Data show mean ± SEM (*P < 0·05, **P < 0·01).

We next assessed the functional ability of mAbs CD43‐6E5 and CD43‐10G7, to induce T‐cell co‐stimulation and T‐cell proliferation. Ligation of CD43 mAbs alone did not induce T‐cell proliferation (see Supplementary material, Fig. S3a). Both CD43 mAbs along with TCR signalling induced proliferation of PB T and CB T cells, but at marginally lower levels, compared with CD28 co‐stimulation (Fig. 2a, and see Supplementary material, Fig. S3b). Co‐stimulation via distinct CD43 epitopes could efficiently activate PB CD4^+^ as well as CD8^+^ T‐cell subsets (see Supplementary material, Fig. S3c). In line with the proliferation data, T_6E5‐act_ and T_10G7‐act_ also expressed various T‐cell activation markers including CD69, CD97 and HLA‐DR at comparable levels to T_CD28‐act_ (Fig. 2b, and see Supplementary material, Fig. S3d). However, some of the other T‐cell activation markers including CD25 and CD71 were differentially regulated. T_6E5‐act_ and T_10G7‐act_ expressed lower levels of CD71 compared with T_CD28‐act_. The T_6E5‐act_ expressed comparable levels of CD25 to T_CD28‐act_, whereas the expression of CD25 was significantly lower on T_10G7‐act_ (Fig. 2b, and see Supplementary material, Fig. S3d).

CD43 has been reported to induce homotypic aggregation in leucocytes, including T cells.^35^, ^36^, ^37^, ^38^ T‐cell homotypic clustering is considered as a hallmark for efficient T‐cell activation *in vitro*. Likewise, PB T‐cell activation using immobilized CD43 mAbs along with TCR signalling induced a homotypic clustering response that was clearly visible after 30 hr (see Supplementary material, Fig. S3e).

### Co‐stimulation via two CD43 epitopes differentially regulate activation of transcription factors

Previous studies have shown that T‐cell co‐stimulation via CD43 induces DNA binding activity of NF‐*κ*B, NFAT and AP‐1 transcription factors.^6^ In assays using Jurkat E6 multi‐channel reporter cells that express both CD43 epitopes, co‐stimulation via CD43‐6E5 induced activation of NF‐*κ*B (Fig. 2c, and see Supplementary material, Fig. S1f). However, NF‐*κ*B activation was weaker compared with CD28 co‐stimulation (Fig. 2c). Co‐stimulation via CD43‐10G7 did not further enhance NF‐*κ*B promoter activity compared with CD3 (Fig. 2c). NFAT reporter activity was comparable upon co‐stimulation via CD28 or via CD43‐6E5 but was lower upon activation via CD43‐10G7 (Fig. 2c). Compared with CD3 alone, AP‐1 promoter activity was further enhanced only upon CD28 co‐stimulation (Fig. 2c).

### Differential regulation of helper T‐cell cytokines via CD43

**Figure 3:**
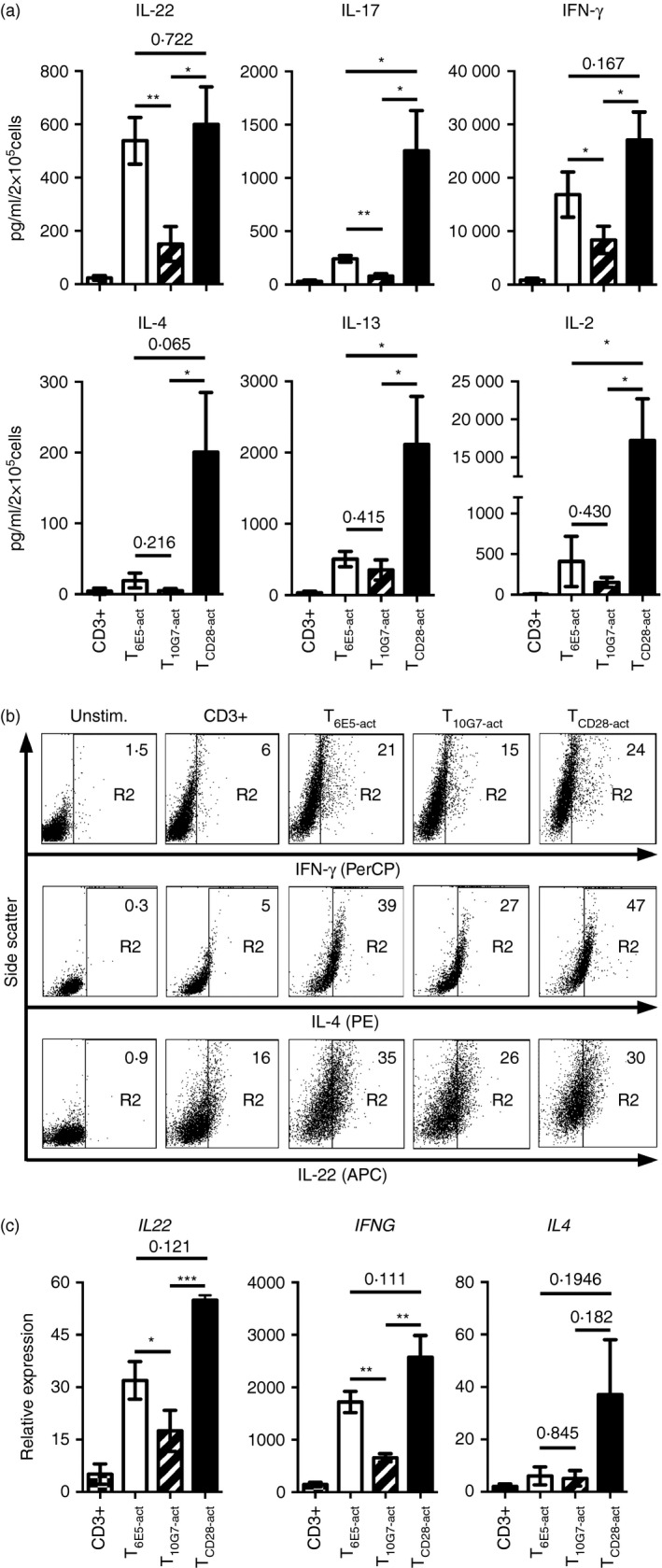
Differential regulation of pro‐inflammatory T‐cell cytokines by CD43. (a) Concentration of T‐cell cytokines measured using Luminex‐based multiplex assay from the supernatants of T cells activated via CD3 (checkered bars), T_6E5‐act_ (open bars), T_10G7‐act_ (striped bars) and T_CD__28‐act_ (black bars) (no. of experiments = 5, no. of donors = 5). (b) Expression of interferon‐*γ* (IFN‐*γ*), interleukin‐4 (IL‐4) and IL‐22 analysed by intracellular cytoplasmic staining in peripheral blood (PB) T cells activated via plate‐bound CD3, CD3/CD43‐6E5, CD3/CD43‐10G7 and CD3/CD28 for 48 hr. Numbers indicate the percentage of positive cells within the gate R2. Gate R2 was set according to the results obtained with unstimulated cells. Gate R1 was set on a live cell population (not shown). Data shown are representative of two independent experiments with two different donors. (c) PB T cells were activated with respective monoclonal antibodies (mAbs) for 30 hr and expression of *IFNG*,*IL4* and *IL22* was analysed by real time PCR in T cells activated via CD3 (checkered bars), T_6E5‐act_ (open bars), T_10G7‐act_ (striped bars) and T_CD__28‐act_ (black bars). Values were normalized to *CD3E* (no. of experiments = 4, no. of donors = 4). (a and c) Data show mean ± SEM (*P < 0·05, **P < 0·01, ***P < 0·001).

T‐cell co‐stimulation via CD43 uses overlapping as well as distinct signalling pathways from CD28 co‐stimulation that in turn may differentially regulate target gene expression in primary human T cells.^9^ To further investigate the effect of CD43 co‐stimulation on subsequent T helper cell function, the production and secretion of various T‐cell cytokines in the supernatants of activated PB T cells was analysed. Similar to T_CD28‐act_, T_6E5‐act_ secreted high levels of IL‐22 and IFN‐*γ* in the cell supernatant. However, compared with T_CD28‐act_, the measured levels of IL‐17 were significantly low in T_6E5‐act_ cell supernatants (Fig. 3a). In contrast to T_6E5‐act_, supernatants of T_10G7‐act_ contained significantly low amounts of IL‐22, IFN‐*γ* and also IL‐17 (Fig. 3a). Compared with T_CD28‐act_, supernatants of T_6E5‐act_ as well as T_10G7‐act_ contained very low levels of IL‐2 and T helper type 2 cytokines including IL‐4 and IL‐13 (Fig. 3a). These results were further confirmed by intracellular cytokine staining (Fig. 3b, and see Supplementary material, Table S2). The analysis of cytokine production at the protein level correlated with induction of mRNA. Results presented in Fig. 3(c) demonstrate that T_10G7‐act_ expressed low levels of *IFNG*,* IL4* and *IL22* mRNA. On the other hand, *IFNG* and *IL22* mRNA levels were high in T_6E5‐act_ similar to T_CD28‐act_. However, T_6E5‐act_ expressed only low levels of *IL4* mRNA. The data suggest that T_6E5‐act_ show substantial overlap with T_CD28‐act_, except for the induction of IL‐4, IL‐13 and IL‐2, whereas induction of various T‐cell signature cytokines is differentially regulated between the two epitopes on CD43 analysed in this study.

### Regulation of anti‐inflammatory cytokines by CD43 co‐stimulation

**Figure 4:**
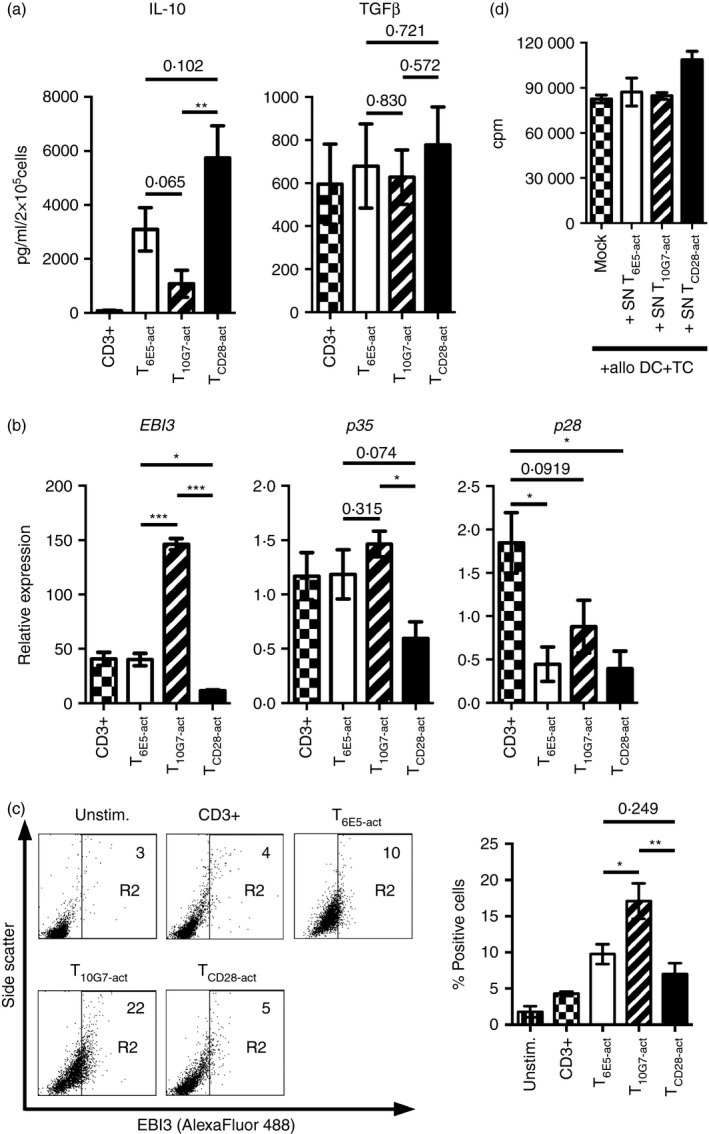
T_10G7‐act_ produce high levels of inhibitory cytokines transforming growth factor‐*β* (TGF‐*β*) and interleukin‐35 (IL‐35) subunit, EBI3. (a) Concentration of IL‐10 and TGF‐*β* from the supernatants of T cells activated via CD3 (checkered bars), T_6E5‐act_ (open bars), T_10G7‐act_ (striped bars) and T_CD__28‐act_ (black bars) analysed by Luminex based multiplex assay (no. of experiments = 5, no. of donors = 5). (b) Expression of *EBI3* and *p35* and *p28* by quantitative PCR in T cells activated by the respective monoclonal antibodies (mAbs) for 30 hr. Values were normalized to *CD3E* (no. of experiments = 4, no. of donors = 4). (c) Expression of EBI3 analysed by intracellular cytoplasmic staining, in peripheral blood (PB) T cells activated via the respective mAbs for 48 hr. Numbers indicate the percentage of positive cells within the gate R2. Gate R2 was set according to the results obtained with unstimulated cells. Gate R1 was set on live cell population (not shown). Data shown are representative of three independent experiments with three different donors. Bar diagram show percentage of EBI3 positive cells (*n* = 3). (a–c) Data show mean ± SEM (*P < 0·05, **P < 0·01, ***P < 0·001). (d) Supernatants of T_6E5‐act_, T_10G7‐act_ and T_CD__28‐act_ collected at day 3, were added to an allogeneic MLR. Data show T‐cell proliferation, measured by [methyl‐^3^H]thymidine incorporation, induced by DC in the presence or absence (Mock) of indicated cell supernatants. Data shown are representative of four independent experiments with four different donors. Data show mean ± SD.

Along with inflammatory cytokines, regulatory cytokines play a crucial role in shaping an effective immune response. Therefore, apart from pro‐inflammatory T‐cell cytokines, the induction of regulatory T‐cell cytokines such as IL‐10 and TGF‐*β* was also analysed. Production of IL‐10 was strongly induced by CD28 co‐stimulation, whereas T_6E5‐act_ secreted moderate levels of IL‐10 (Fig. 4a). The TGF‐*β* was similarly regulated by all the three co‐stimulations tested (Fig. 4a). In addition, the expression of IL‐35 subunits, *EBI3* and *p35* was also analysed. T‐cell co‐stimulation via CD43‐10G7 induced significantly higher expression of *EBI3* mRNA compared with T cell co‐stimulation either via CD43‐6E5 or CD28 (Fig. 4b). The higher expression of EBI3 protein in T_10G7‐act_ was further confirmed by intracellular staining (Fig. 4c). The expression of *p35* was also slightly up‐regulated in T_10G7‐act_ compared with either T_6E5‐act_ or T_CD28‐act_ (Fig. 4b). The IL‐35 subunit EBI3 can dimerize with p28 to form IL‐27.^39^ The expression levels of *p28* were in fact lower upon co‐stimulation with all three stimuli tested here, compared with stimulation via CD3 alone (Fig. 4b).

Interleukin‐35 is regarded as an inhibitory cytokine; therefore supernatants of activated PB T cells were tested for inhibitory effect in an allogeneic MLR. However, we could not observe a soluble factor mediated inhibition of T‐cell proliferation (Fig. 4d).

### T‐cell co‐stimulation via CD43‐10G7 induces a hypo‐proliferative state

**Figure 5:**
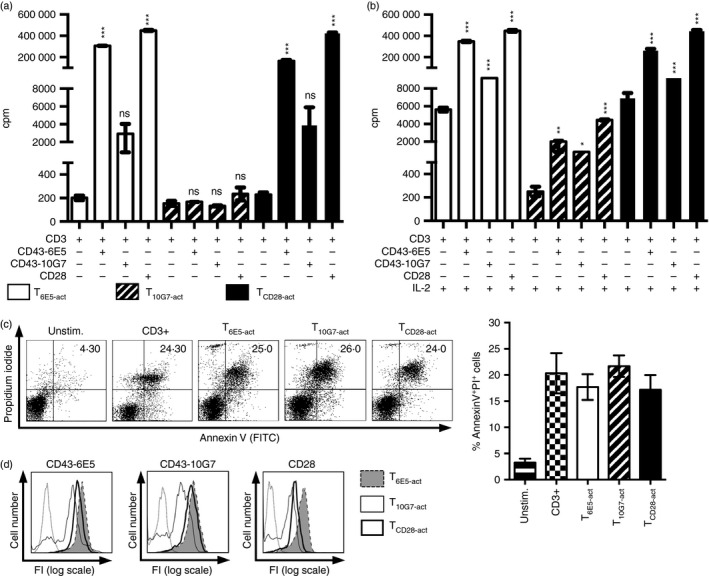
T_10G7‐act_ acquire a hypo‐proliferative state. (a) T_6E5‐act_ (open bars), T_10G7‐act_ (striped bars) and T_CD__28‐act_ (black bars) were re‐stimulated with the indicated co‐stimulus. T‐cell proliferation was measured by analysing [methyl‐^3^H]thymidine incorporation. (b) As in (a), but with the addition of exogenous interleukin‐2 (IL‐2; 20 U/ml). (a, b) Data show mean ± SD. (*P < 0·05, **P < 0·01, ***P < 0·001) (c) Data show Annexin V (FITC) and propidium iodide staining of unstimulated T cells, T cells activated via CD3, T_6E5‐act_, T_10G7‐act_ and T_CD__28‐act_. Gate was set on whole cell population. Quadrant marker was set according to results obtained with unstained cells (not shown). Numbers indicate percentage of positive cells within upper right quadrant. Data shown are representative of five independent experiments with five different donors. Bar diagram show percentage of Annexin V/PI‐positive cells. Data show ± SEM (*n* = 5). (d) Data show expression of CD43‐6E5, CD43‐10G7 and CD28 on T_6E5‐act_ (grey filled histograms), T_10G7‐act_ (thin open histograms) and on T_CD__28‐act_ (thick open histograms) analysed before re‐stimulation. Reactivity profile of isotype control is also shown (open dotted histograms). Gate was set on live cell population (not shown). (a, b and d) Data shown are representative of two independent experiments with two different donors.

In the next set of experiments, we analysed the ability of T cells activated in the presence of CD43 co‐stimulatory signals to respond to re‐stimulation. In contrast to T_6E5‐act_ or T_CD28‐act_ that responded efficiently to re‐stimulation, T_10G7‐act_ showed reduced proliferative responses irrespective of whether CD3/CD43‐6E5, CD3/CD43‐10G7 or CD3/CD28 was used as the secondary stimulus (Fig. 5a). As opposed to T_6E5‐act_ and T_CD28‐act_, the addition of exogenous IL‐2 had no effect on re‐stimulation of T_10G7‐act_ with CD3 alone (Fig. 5a,b). Exogenous IL‐2 could restore the proliferation of T_10G7‐act_ in the presence of a co‐stimulatory signal during re‐stimulation; however, not as effectively as in the case of _T6E5‐act_ and T_CD28‐act_ (Fig. 5b). The reduced proliferative capacity of T_10G7‐act_ seemed not to be due to increased T‐cell death, as indicated by Annexin V and propidium iodide staining (Fig. 5c). Furthermore, the reduced proliferation response was neither due to down‐modulation of co‐receptors during the first round of activation. We analysed the expression of CD43 epitopes recognized by mAbs CD43‐6E5, CD43‐10G7 and of CD28 on T_6E5‐act_, T_10G7‐act_ and T_CD28‐act_ (Fig. 5d). Though all three mAbs showed slightly reduced binding to T_10G7‐act_, this did not correlate with the extent of poor proliferative response induced upon re‐stimulation (Fig. 5a,b, d).

Hence, in contrast to the T‐cell co‐stimulation via the CD43‐6E5‐defined epitope or via CD28, T‐cell co‐stimulation via the CD43‐10G7‐defined epitope induces a deep hypo‐proliferative state in T cells.

### T_10G7‐act_ acquire an inhibitory function

So far, T_10G7‐act_ exhibited properties similar to many different subsets of inhibitory T cells, such as lower levels of IFN‐*γ*, IL‐2, IL‐4 and IL‐22 but up‐regulation of IL‐35 cytokine subunits, EBI3 and p35 and higher production of TGF‐*β*.^40^, ^41^, ^42^

**Figure 6:**
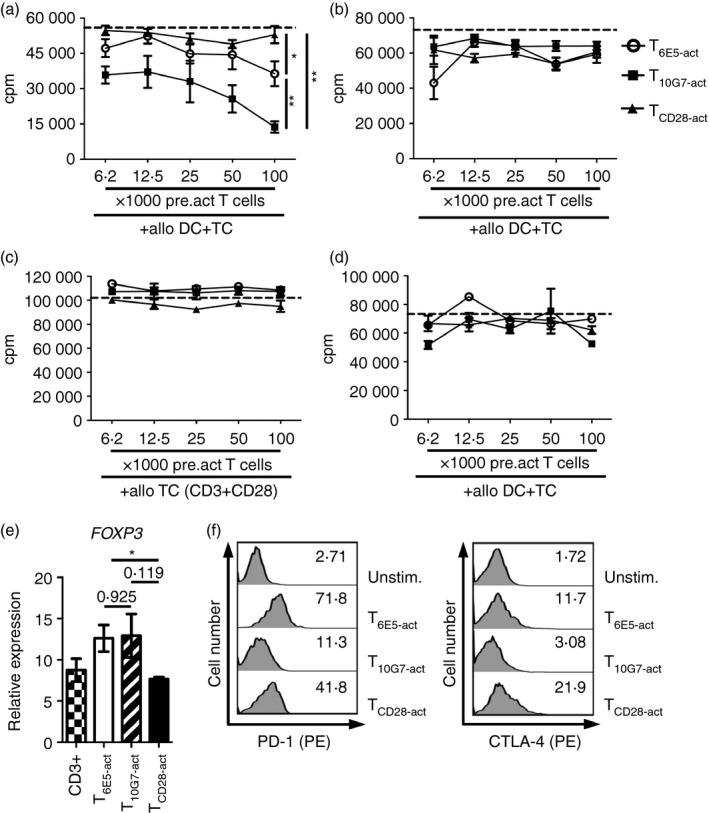
T_10G7‐act_ acquire an inhibitory function. (a) Purified responder T cells (1 × 10^5^ cells) were stimulated with allogeneic dendritic cells (DC) (5 × 10^4^ cells) alone (dashed line) or in the presence of graded number of irradiated T_6E5‐act_, T_10G7‐act_ or T_CD__28‐act_. (b) As in (a), but pre‐activated T cells were fixed with 1% formaldehyde, before being added to an allogeneic mixed lymphocyte reaction (MLR). Data are representative of two independent experiments with two different donors. (c) As in (a), but irradiated T cells were added to responder T cells (1 × 10^5^ cells) activated via CD3/CD28 monoclonal antibodies (mAbs). Data are representative of three independent experiments with three different donors. (d) As in (a), but allogeneic DC were also irradiated before being added to an MLR. Data are representative of two independent experiments with two different donors. (b–d) Data show mean ± SD. (e) Expression of *FOXP3* by quantitative PCR in peripheral blood (PB) T cells activated for 30 hr via respective immobilized mAbs. Values were normalized to *CD3E*. (a and e) Data show mean ± SEM (*P < 0·05, **P < 0·01) (no. of experiments = 3, no. of donors = 3). (f) Data show expression of PD‐1 (CD279) and CTLA‐4 (CD152) on unstimulated T cells, T_6E5‐act_, T_10G7‐act_ and T_CD__28‐act_ analysed by flow cytometry_._ Numbers indicate percentage of positive cells. Gate was set on live cell population (not shown). Data are representative of two independent experiments with two different donors.

To further analyse the functional properties of these cells, T‐cell suppression assays were performed. For this, irradiated T_6E5‐act_, T_10G7‐act_ and T_CD28‐act_ were added to an allogeneic MLR. T_10G7‐act_ inhibited DC‐induced T‐cell proliferation in an MLR in a dose‐dependent manner (Fig. 6a). T_10G7‐act_ were as efficient as regulatory T cells in their suppressive capacity (see Supplementary material, Fig. S4a). Compared with T_CD28‐act_, high numbers of T_6E5‐act_ seem to exert an inhibitory effect. However, with T_10G7‐act_ such inhibition could be achieved when almost 10 times fewer cells were used (Fig. 6a). Prior fixation of T_10G7‐act_ cells with formaldehyde reversed their inhibitory function (Fig. 6b).

Furthermore, T_10G7‐act_ could not inhibit T‐cell proliferation when added to allogeneic T cells activated via plate‐bound CD3/CD28 mAb in the absence of APC (Fig. 6c). Hence, the data suggest a crucial role of DC in the inhibition of responder T cells. The suppressive effect of T_10G7‐act_ could also be abrogated when DC were irradiated before being added to an allogeneic MLR (Fig. 6d). This inhibitory effect was not restricted to the presence of DC, as T_10G7‐act_ also exhibited an inhibitory effect when monocytes or monocyte‐derived macrophages were used as APC in an allogeneic MLR (data not shown).

FOXP3 is a forkhead family transcription factor important for the development and function of natural regulatory T cells.^43^ Therefore, expression of FOXP3 was analysed by quantitative PCR as well as by intracellular staining. FOXP3 expression did not differ between different CD43 co‐stimulations, suggesting that the suppressive function of T_10G7‐act_ is not directly related to the expression of FOXP3 (Fig. 6e, and see Supplementary material, Fig. S4b). Activation‐induced transient expression of FOXP3 in T effector cells has been reported before and has not necessarily been associated with suppressive function of T cells in humans.^22^, ^44^, ^45^ Expression of cell surface molecules such as PD‐1 (CD279) and CTLA‐4 (CD152) has been previously linked with regulatory function in T cells.^46^, ^47^, ^48^ However, as analysed by flow cytometry, expression of PD‐1 (CD279) as well as CTLA‐4 (CD152) was in fact lower on T_10G7‐act_ compared with T_6E5‐act_ or T_CD28‐act_ (Fig. 6f).

### The inhibitory T_10G7‐act_ cells do not alter the accessory molecule repertoire on DC

**Figure 7:**
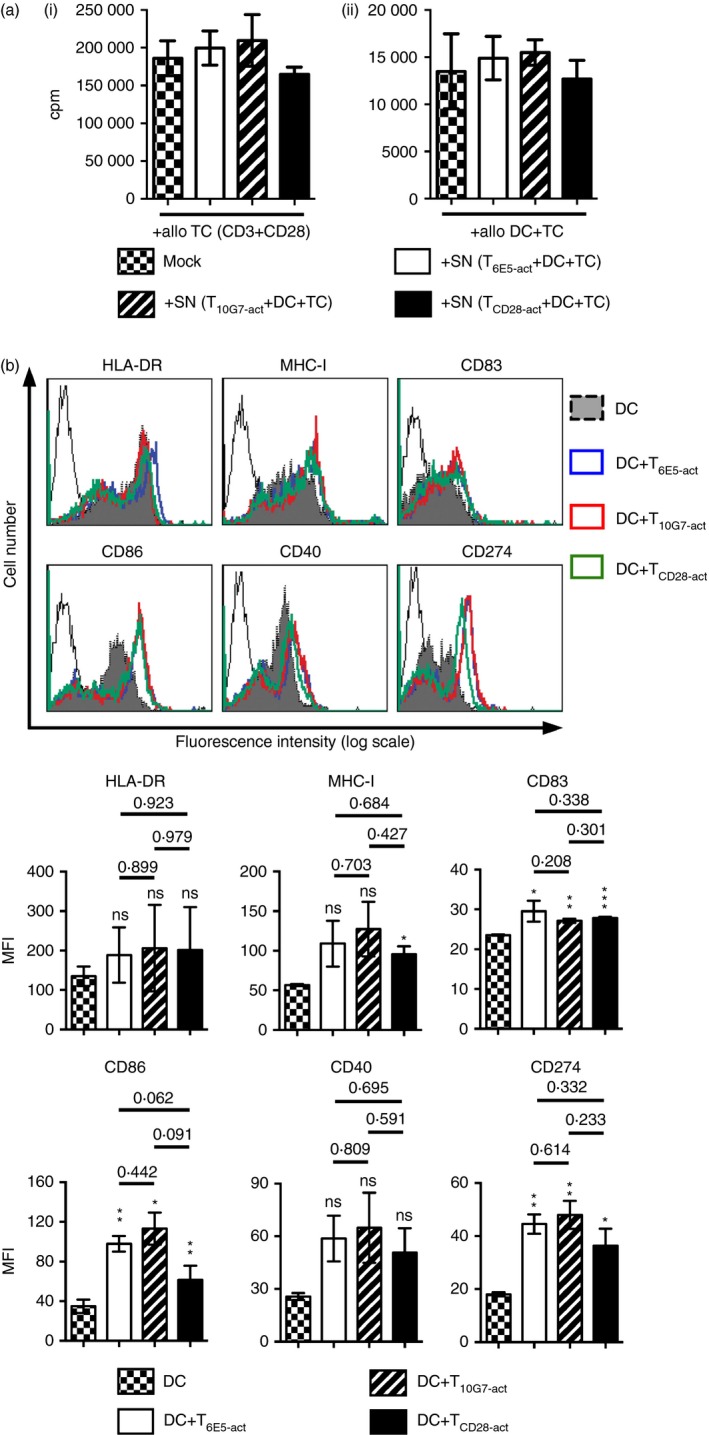
Inhibitory T_10G7‐act_ do not alter accessory molecule repertoire on dendritic cells (DC). (a) Supernatants from an allogeneic mixed lymphocyte reaction (MLR) with irradiated T_6E5‐act_, T_10G7‐act_ and T_CD__28‐act_ collected at day 5 were added to purified T cells (2 × 10^5^ cells) activated via CD3/CD28 monoclonal antibodies (mAbs) (i) or to an allogeneic MLR (ii). T‐cell proliferation was measured by analysing [methyl‐^3^H]thymidine incorporation. Data show mean ± SD. Data are representative of three independent experiments with three different donors. (b) Cell surface marker expression on DC (grey filled histograms), DC co‐cultured with T_6E5‐act_ (blue line), T_10G7‐act_ (red line) and T_CD__28‐act_ (green line). DC were co‐cultured with pre‐activated T cells for 48 hr and were analysed by flow cytometry. Overlays show reactivity profile of various mAbs including isotype control (black line). Gate was set on CD1a^+^ cell population (not shown). Data are representative of three independent experiments with three different donors. Bar diagrams show mean fluorescence intensity (MFI) of indicated cell surface receptors on DC (checkered bars), DC co‐cultured with T_6E5‐act_ (open bars), T_10G7‐act_ (striped bars) and T_CD__28‐act_ (black bars). Data show ± SEM (*n* = 3) (*P < 0·05, **P < 0·01, ***P < 0·001).

The finding that the inhibitory effect of T_10G7‐act_ was observed only in the presence of APC, prompted us to analyse whether a soluble factor secreted by DC co‐cultured with T_10G7‐act_ might be responsible for the inhibitory effect. However, results shown in Fig. 7(a) demonstrate that supernatant from an MLR with pre‐activated PB T cells had no inhibitory effect on the proliferation of responder PB T cells.

Next, the expression of various cell surface receptors on DC co‐cultured in an allogeneic MLR with T_6E5‐act_, T_10G7‐act_ or T_CD28‐act_ was analysed. DC co‐cultured with T_10G7‐act_ showed comparable expression of MHC‐I, HLA‐DR to DC co‐cultured with either T_6E5‐act_ or T_CD28‐act_. The same is true for DC maturation markers such as CD83 or for co‐stimulatory molecules such as CD86 and CD40 (Fig. 7b). Interestingly, the inhibitory receptor B7‐H1 (CD274) was not altered on DC co‐cultured with T_10G7‐act_ (Fig. 7b).

### T_10G7‐act_ induce stable heterotypic clustering with DC to constrain activation of responder T cells

**Figure 8:**
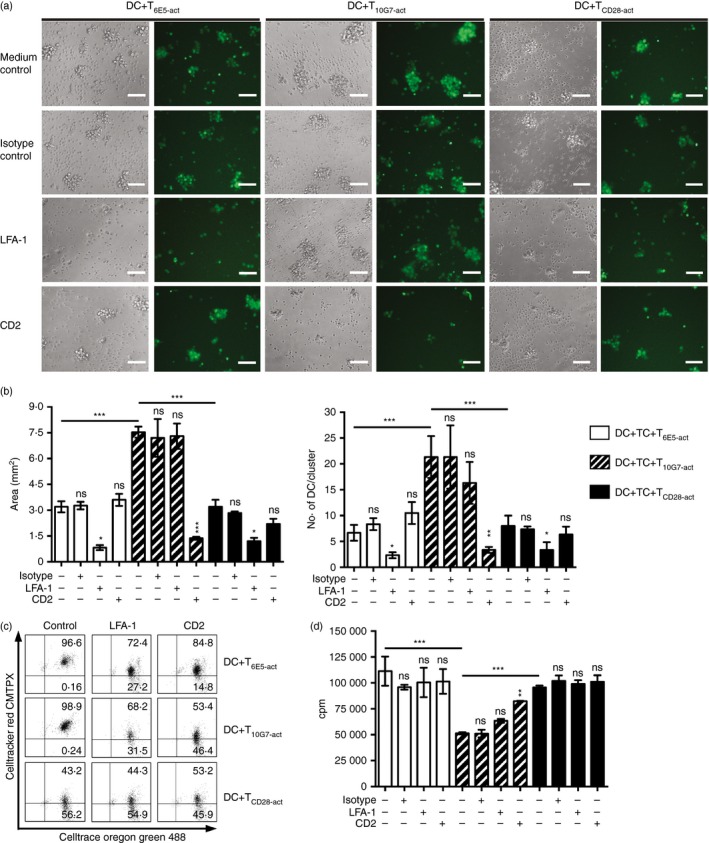
T_10G7‐act_ form stable clusters with dendritic cells (DC), via CD2/CD58. (a) DC labelled with CellTrace^™^ Oregon Green^®^ 488 co‐cultured with either irradiated (unlabelled) T_6E5‐act_, T_10G7‐act_, T_CD__28‐act_ or cells pre‐treated with respective blocking monoclonal antibodies (mAbs) before irradiation. Original magnification: 10 ×; scale bar: 50 μm. Data are representative of three independent experiments with three different donors. (b) Bar diagrams show area of cluster and number of DC per cluster (CellTrace^™^ Oregon Green^®^ 488‐positive cells). DC were co‐cultured with irradiated T_6E5‐act_ (open bars), T_10G7‐act_ (striped bars) and T_CD__28‐act_ (black bars). Before irradiation, T_6E5‐act_, T_10G7‐act_ and T_CD__28‐act_ were pre‐treated with the indicated mAbs. (c) Quantitative analysis of DC–T‐cell clustering by flow cytometry. DC were labelled with CellTrace^™^ Oregon Green^®^ 488; irradiated pre‐activated T cells were labelled with CellTracker^™^ Red CMTPX. Where indicated, pre‐activated T cells were pre‐treated with respective blocking mAbs. Double‐positive cells were counted as T cells and DC cluster. Numbers indicate percentage of cells within the quadrant. For gating strategy please see Supplementary material, Fig. S6. Data are representative of two independent experiments with two different donors. (d) T_6E5‐act_ (open bars), T_10G7‐act_ (striped bars) or T_CD__28‐act_ (black bars) were pre‐treated with the indicated blocking mAbs, before irradiation and were then added to allogeneic DC and responder T cells. Proliferation of responder T‐cell was measured by analysing [methyl‐^3^H]thymidine incorporation at day 5. (b, d) Data show mean ± SD. Data are representative of three independent experiments with three different donors (*P < 0·05, **P < 0·01, ***P < 0·001).

Dendritic cells co‐cultured with irradiated T_10G7‐act_ showed characteristic cluster formations, compared with DC co‐cultured with irradiated T_6E5‐act_ or T_CD28‐act_ as shown by the area of each cluster and the number of DC per cluster (Fig. 8a,b). Heterotypic aggregate formation was found to be an active process. Neither irradiated T cells co‐stimulated via plate‐bound CD3/CD43‐10G7 mAbs (see Supplementary material, Fig. S5a), nor irradiated DC co‐cultured with irradiated T_10G7‐act_ (see Supplementary material, Fig. S5b) were able to form aggregates. Likewise, regulatory T cells have been reported to physically hinder the interaction of DC with responder T cells by forming large aggregates.^49^, ^50^, ^51^ Hence, T_10G7‐act_‐induced stable heterotypic clustering may contribute to the inhibitory effect of T_10G7‐act_ on the accessory function of DC in co‐culture experiments.

As an underlying mechanism, T_10G7‐act_‐induced heterotypic clustering was found to be mainly CD2 dependent, whereas interaction of T_6E5‐act_ or T_CD28‐act_ with co‐cultured DC was LFA‐1 (CD11a/CD18) dependent (Fig. 8a–c). Heterotypic interaction of T_CD28‐act_ with co‐cultured DC was weaker compared with T_6E5‐act_ and T_10G7‐act_, as shown by a higher percentage of single‐positive DC in T_CD28‐act_ (56·2%) control treated cells compared with T_6E5‐act_ (0·16%) and T_10G7‐act_ (0·24%) (Fig. 8c).

Further, pre‐treatment of T_10G7‐act_ with a CD2 blocking mAb could abolish the inhibitory effect of T_10G7‐act_ and restore proliferation of responder T cells. The pre‐treatment of T_10G7‐act_ with anti LFA‐1 blocking mAb could slightly restore proliferation compared with isotype control but not as effectively as pre‐treatment with CD2 (Fig. 8d).

## Discussion

Signalling via accessory cell surface receptors plays an essential role in the induction, tuning and regulation of T‐cell activation and function.^52^ A plethora of such co‐stimulatory receptors have been identified, which conventionally provide either positive or negative signals to T cells. CD43 is one of the most abundant cell surface receptors on human T cells. Yet, the functional role of CD43 on T cells is still controversial, with several studies reporting opposing roles of CD43 in T‐cell function.^4^, ^5^, ^6^, ^8^, ^9^, ^13^, ^14^, ^17^, ^18^ Here, we demonstrate that targeting of distinct epitopes on CD43 can decide the subsequent fate of T‐cell function.

This observation was made with two well‐defined CD43 mAbs (6E5, 10G7) directed against two non‐overlapping binding sites, expressed on both isoforms of CD43 (Fig. 1a, and see Supplementary material, Fig. S1a,b,e).^19^, ^20^ CD43‐6E5 mAb shares a similar epitope to CD43 mAb MEM‐59.^19^ Co‐stimulation via MEM‐59 along with TCR signalling could efficiently induce T‐cell activation.^6^, ^53^ Previous studies have demonstrated that targeting of CD43 with mAbs CD43‐6E5 and CD43‐10G7 induces aggregation and oxidative burst formation in neutrophils.^37^ Nonetheless, epitope recognized by mAb CD43‐6E5 but not CD43‐10G7 was found to be involved in T‐cell conjugate formation with APC.^16^ We observed that both mAbs could potently activate T cells in the presence of TCR signalling, inducing a strong proliferative response in T cells including CB T cells and CD4^+^ as well as CD8^+^ T‐cell subsets (Fig. 2a, and see Supplementary material, Fig. S3a–c). T‐cell activation via CD43 was accompanied by the expression of classical T‐cell activation markers such as CD69 and induction of homotypic clustering – a hallmark of T‐cell activation *in vitro* (Fig. 2b, and see Supplementary material, Fig. S3d,e).

Mattioli *et al.,* have previously suggested that CD28 and CD43 may use different as well as overlapping signalling pathways. As a result, T‐cell co‐stimulation via CD43 may trigger expression of similar as well as different target genes compared with CD28 co‐stimulation, e.g. the expression of the important T‐cell cytokine gene *IL2*.^9^ Similarly, we observed that T_6E5‐act_ and T_10G7‐act_ produced low levels of IL‐2 compared with T_CD28‐act_. Various studies have reported that cytokine production and T‐cell proliferation are autonomously regulated upon T‐cell activation.^54^, ^55^ Yet, such low amounts of IL‐2 produced upon co‐stimulation via CD43 could be sufficient to promote T‐cell proliferation (Figs 2a, 3a).^56^ Compared with T_CD28‐act_, T_6E5‐act_ produced similarly high levels of IFN‐*γ* and IL‐22 but only low amounts of IL‐2, IL‐4, IL‐13 and IL‐17. Yet, the CD43 mAbs 6E5 and 10G7 exerted polarizing effects on T cells (Fig. 3). We found that T_10G7‐act_ synthesized low amounts of all analysed cytokines except of the inhibitory cytokines TGF‐*β* and IL‐35 subunit EBI3 (Figs 3 and 4a–c). Polarizing effects observed upon ligation of our two CD43 mAbs, seem not to be dependent on recognition of specific CD43 isoforms. Downstream of T‐cell co‐stimulation via different CD43 epitopes, CD4^+^ and CD8^+^ T cells showed similar functional responses like bulk T cells. Both CD4^+^ and CD8^+^ T cells, activated via CD43‐6E5 expressed higher levels of *IFNG* but lower levels of *EBI3* mRNA, compared with their counterparts activated via CD43‐10G7 (see Supplementary material, Fig. S7).

Downstream of co‐stimulation, signal transduction through CD43 has been reported to induce DNA binding activity of NF‐*κ*B, NFAT and AP‐1 transcription factors when CD43 is cross‐linked with mAbs.^6^, ^7^, ^8^ In our test system co‐stimulation via CD43 did not induce activation of AP‐1 of transcription factor (Fig. 2c). T‐cell stimulation via CD43‐6E5 mAb induced activation of NFAT and NF‐*κ*B. However, co‐stimulation via mAb CD43‐10G7 could only induce activation of NFAT (Fig. 2c). Following T‐cell stimulation, activation of NFAT in the absence of NF‐*κ*B and AP‐1 activation leads to anergy.^57^ This mechanism could explain the hypo‐proliferative phenotype of T_10G7‐act_ as opposed to T_6E5‐act_ seen upon re‐stimulation (Figs 2c, 5a,b). In T cells, this anergic state has also been observed for low‐strength T‐cell activation in the presence of co‐stimulation.^58^ The mAb CD43‐10G7 showed similar binding affinity and kinetics as CD43‐6E5 (see Supplementary material, Fig. S1c,d). T‐cell co‐stimulation via CD43‐10G7 could efficiently induce T‐cell proliferation similar to co‐stimulation via CD43‐6E5, at levels higher than CD3 alone (Fig. 2a, and see Supplementary material, Fig. S3a–c). Compared with T cells activated via CD3 alone T_10G7‐act_ also showed higher induction of IFN‐*γ*, IL‐22 and IL‐10, but at levels lower than other co‐stimuli tested (Figs 3 and 4a, and see Supplementary material, Fig. S7). It will be interesting to elucidate in future studies, whether a distinct signalling pathway or a putative low strength of T‐cell activation upon CD43 cross‐linking with CD43‐10G7 compared to CD43‐6E5 is responsible for the observed co‐stimulatory functions of CD43‐10G7 mAb.

Interestingly, T_10G7‐act_ acquired a prominent inhibitory function in contrast to T_6E5‐act_ or T_CD28‐act_. The suppressive function of T_10G7‐act_ was not mediated via a soluble factor but was dependent on cell–cell contacts. We could also show that T_10G7‐act_ did not directly act on responder T cells. Instead T_10G7‐act_ exhibited their suppressor function via APC such as DC (Fig. 6a,c,d). CD4^+^ CD25^+^ FOXP3^+^ natural regulatory T cells have also been previously reported to route their suppressor function via APC.^59^ One of the mechanisms includes modulating DC to produce immunosuppressive factors such as indolamine 2,3‐dioxygenase.^60^, ^61^ However, in our experiments, the supernatants from DC co‐cultured with T_10G7‐act_ were not inhibitory (Fig. 7a). Another reported mechanism is via CTLA‐4 that results in the suppression of CD80 or CD86 expression.^59^, ^62^ We did not observe such reduced expression of CD80 or CD86 on DC co‐cultured with T_10G7‐act_ (Fig. 7b). Indeed, T_10G7‐act_ expressed lower levels of inhibitory receptors including CTLA‐4 (CD152) and PD‐1 (CD279) than T_6E5‐act_ or T_CD28‐act_ (Fig. 6f). The third mechanism of inhibition is by promoting a long stable interaction of regulatory T cells with DC mediated via various molecules including LFA‐1/ICAM‐1, CD2/CD58, neuropillin‐1 and thereby limiting the activation of responder T cells.^49^, ^50^, ^51^, ^59^ Additionally, a cross‐talk between CD2 and other co‐stimulatory receptors like CD43 and CD28 that facilitates T‐cell conjugate formation and T‐cell activation, respectively, has been reported.^16^, ^63^ Indeed, we have also observed a characteristic large aggregate formation when DC were co‐cultured with T_10G7‐act_ that is primarily mediated via CD2. On the other hand, interaction of T_6E5‐act_ with DC was mediated via LFA‐1 (CD11a/CD18) (Fig. 8a–c). Blocking LFA‐1 (CD11a/CD18) on T_10G7‐act_ showed marginal effect on proliferation, whereas blocking CD2 on T_10G7‐act_ abolished the suppressive effect and restored the proliferation of responder T cells (Fig. 8d).

CD43 is a conserved, sialylated glycoprotein with an elongated extracellular domain.^2^, ^3^ Multiple ligands have been described for CD43: ICAM‐1 (CD54), MHC‐I, Siglec‐1 (CD169), galectin‐1, E‐selectin and albumin.^16^, ^64^, ^65^, ^66^, ^67^, ^68^ The mAb CD43‐6E5‐defined epitope is involved in mediating the binding of MHC‐I molecules, an event that further strengthens the interaction of APC with T cells.^16^, ^19^ For the epitope defined by mAb CD43‐10G7, no potential ligand has been described so far. One candidate for this binding site might be Siglec‐1. We have recently reported that Siglec‐1 on DC contributes to the induction of EBI3 expression and IL‐35 production in co‐cultured T cells.^22^ In this study we have observed that T_10G7‐act_ have a preferential bias to produce IL‐35, so it is tempting to speculate that Siglec‐1 might be a ligand for the CD43‐10G7‐defined epitope. The ability of the natural ligand to modulate CD43 expression might be an important feature of CD43 function. Depending on the test system and cell type used, cross‐linking of CD43 with mAb or phorbol activation has been reported to induce CD43 internalization or proteolytic cleavage in leucocytes.^34^, ^69^, ^70^ Such activation‐induced proteolytic cleavage of CD43 when observed in T cells, is associated with T‐cell stimulation and homeostasis.^69^ Hence, results presented in Fig. 1(b) offer a potential mechanism that cross‐linking of CD43‐6E5, in contrast to CD43‐10G7, allows the down‐modulation of CD43 from the cell surface and that in the absence of CD43 processing T cells acquire a regulatory function. However, these possibilities need to be analysed in more detail in future studies.

Taken together, our data suggests a unique role of CD43 in polarization of T‐cell immunity. These findings could provide an explanation for various contradictory roles of CD43 in T‐cell functions that have been previously reported.

## Disclosures

The authors declare no financial and commercial conflict of interest.

## Supporting information


**Figure S1.** Reactivity profile of CD43 monoclonal antibodies CD43‐6E5 and CD43‐10G7 on human T cells.

**Figure S2.** Down‐modulation of CD43 surface expression by CD43‐6E5 requires T‐cell receptor signalling.

**Figure S3.** CD4^+^, CD8^+^ peripheral blood T and cord blood T‐cell stimulation upon engagement of CD43 monoclonal antibodies.

**Figure S4.** T_10G7‐act_ acquire FOXP3‐independent suppressive function.

**Figure S5.** Heterotypic interaction of dendritic cells with pre‐activated T cells is an active process.

**Figure S6.** Gating strategy for flow cytometry analysis of cluster formation between dendritic cells and T cells.

**Figure S7.** The downstream effect of CD43 co‐stimulation is similar in CD4^+^ and CD8^+^ T cells.

**Table S1.** Real time PCR primer sequences.

**Table S2.** Cytokine profile of _T6E5‐act_, T_10G7‐act_ and T_CD28‐act_.
