# Supplementary figures and images for: Engagement of distinct epitopes on CD43 induces different co‐stimulatory pathways in human T cells
Source: Immunology. 2016 Aug 16;149(3):280–96. doi: 10.1111/imm.12642 (PMC5046061; doi:10.1111/imm.12642)

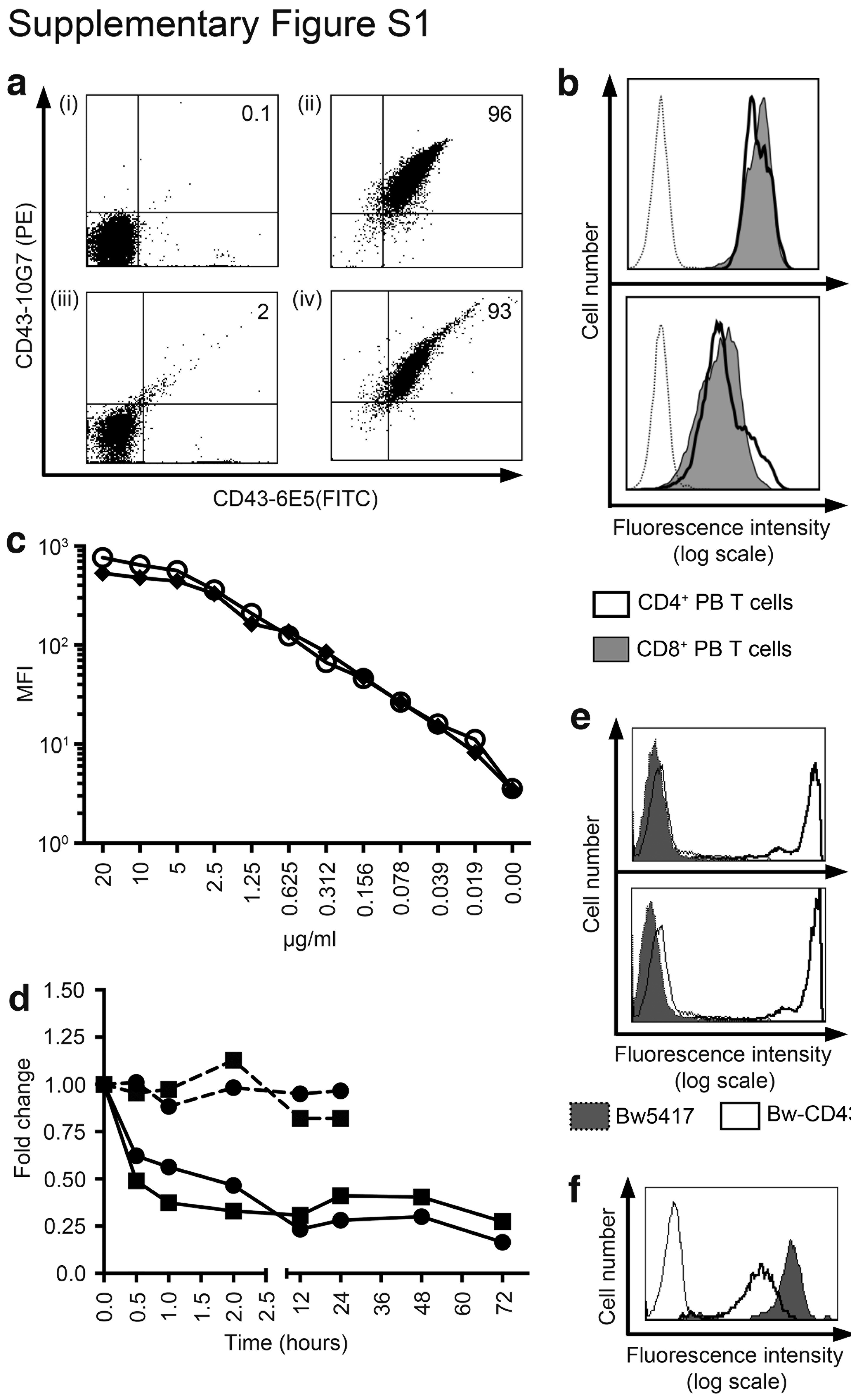

Supplement: Supplementary file 1 — Figure S1. Reactivity profile of CD43 monoclonal antibodies CD43‐6E5 and CD43‐10G7 on human T cells. [file IMM-149-280-s001.tif]

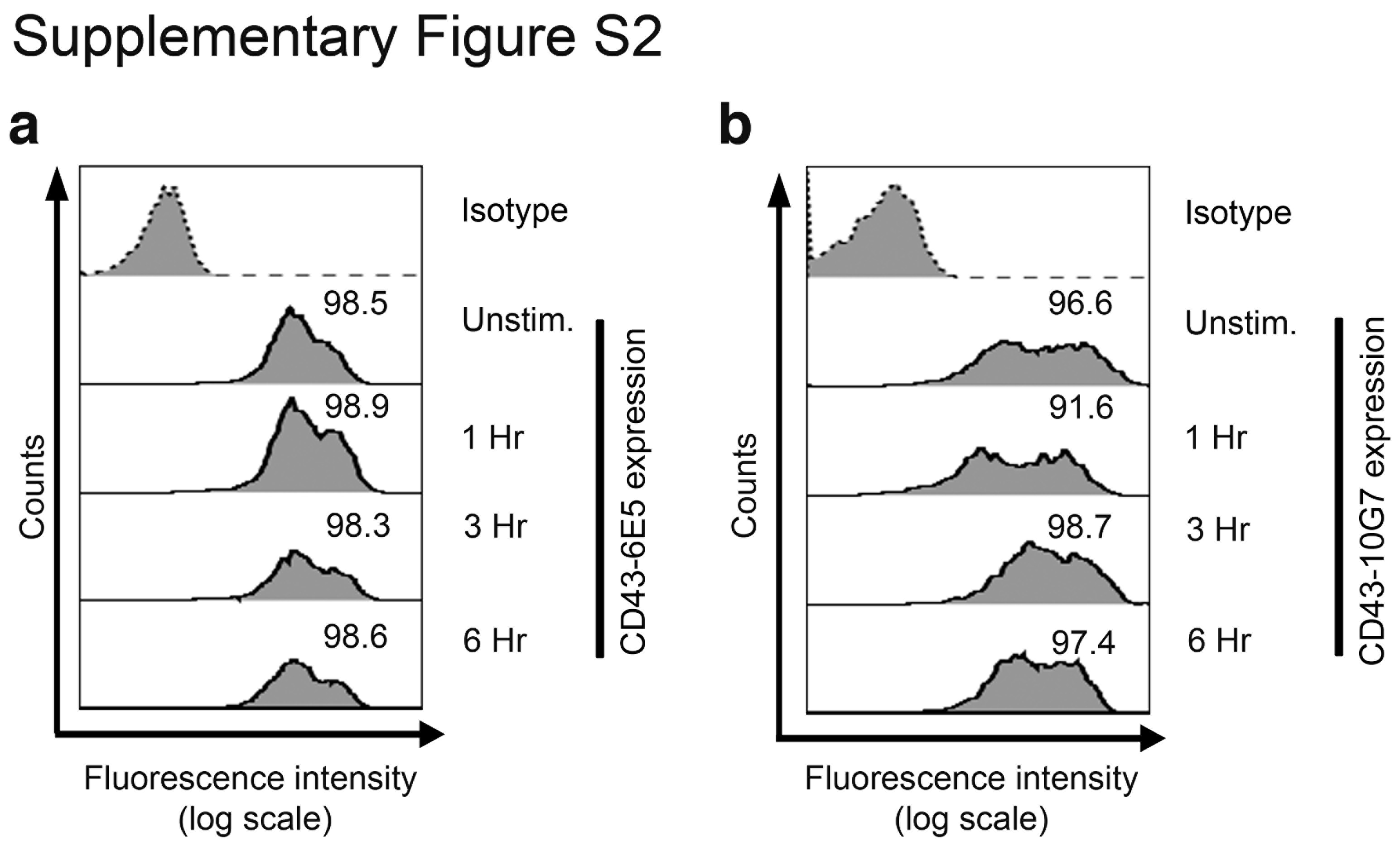

Supplement: Supplementary file 2 — Figure S2. Down‐modulation of CD43 surface expression by CD43‐6E5 requires T‐cell receptor signalling. [file IMM-149-280-s002.tif]

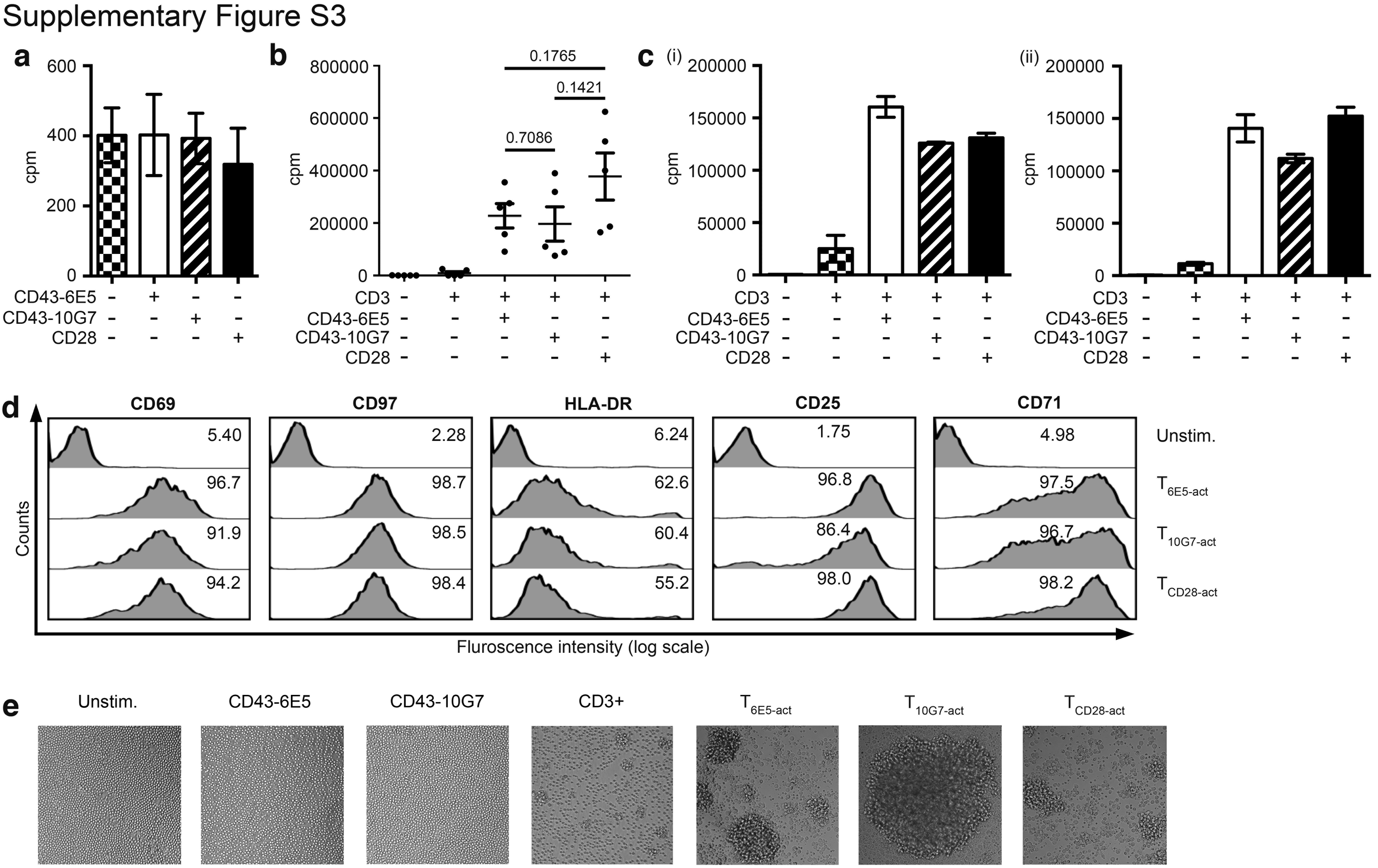

Supplement: Supplementary file 3 — Figure S3. CD4+, CD8+ peripheral blood T and cord blood T‐cell stimulation upon engagement of CD43 monoclonal antibodies. [file IMM-149-280-s003.tif]

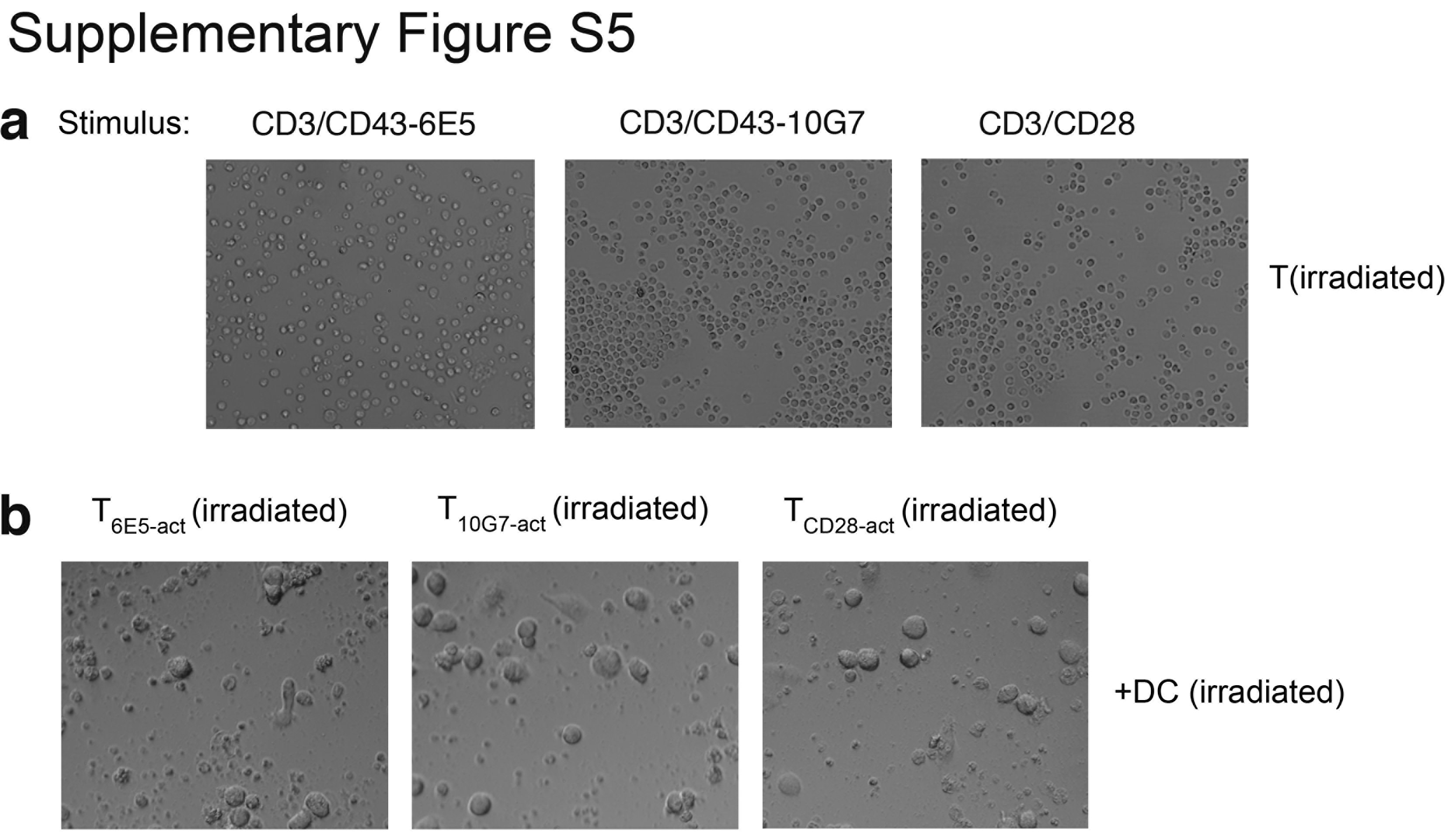

Supplement: Supplementary file 5 — Figure S5. Heterotypic interaction of dendritic cells with pre‐activated T cells is an active process. [file IMM-149-280-s005.tif]

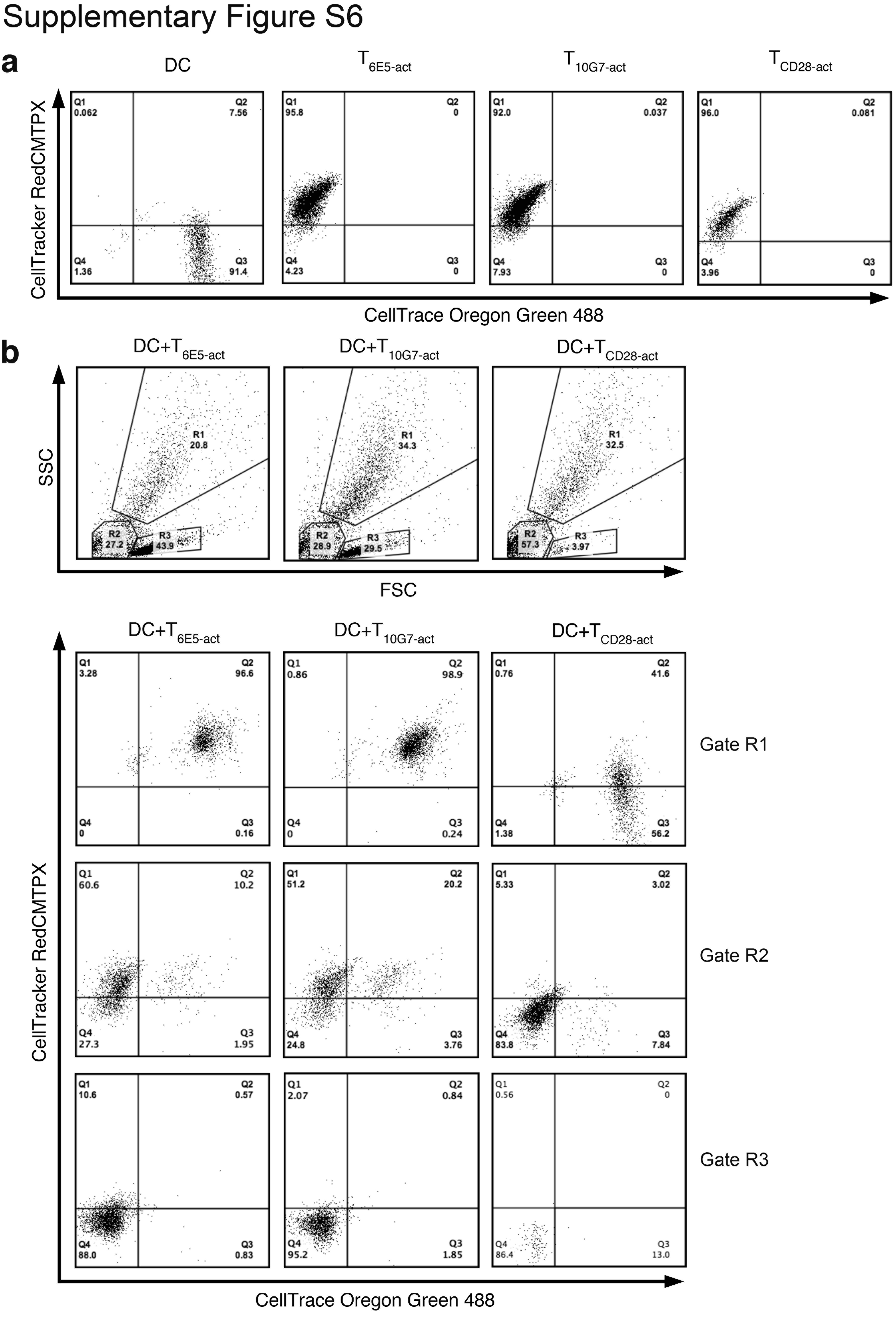

Supplement: Supplementary file 6 — Figure S6. Gating strategy for flow cytometry analysis of cluster formation between dendritic cells and T cells. [file IMM-149-280-s006.tif]

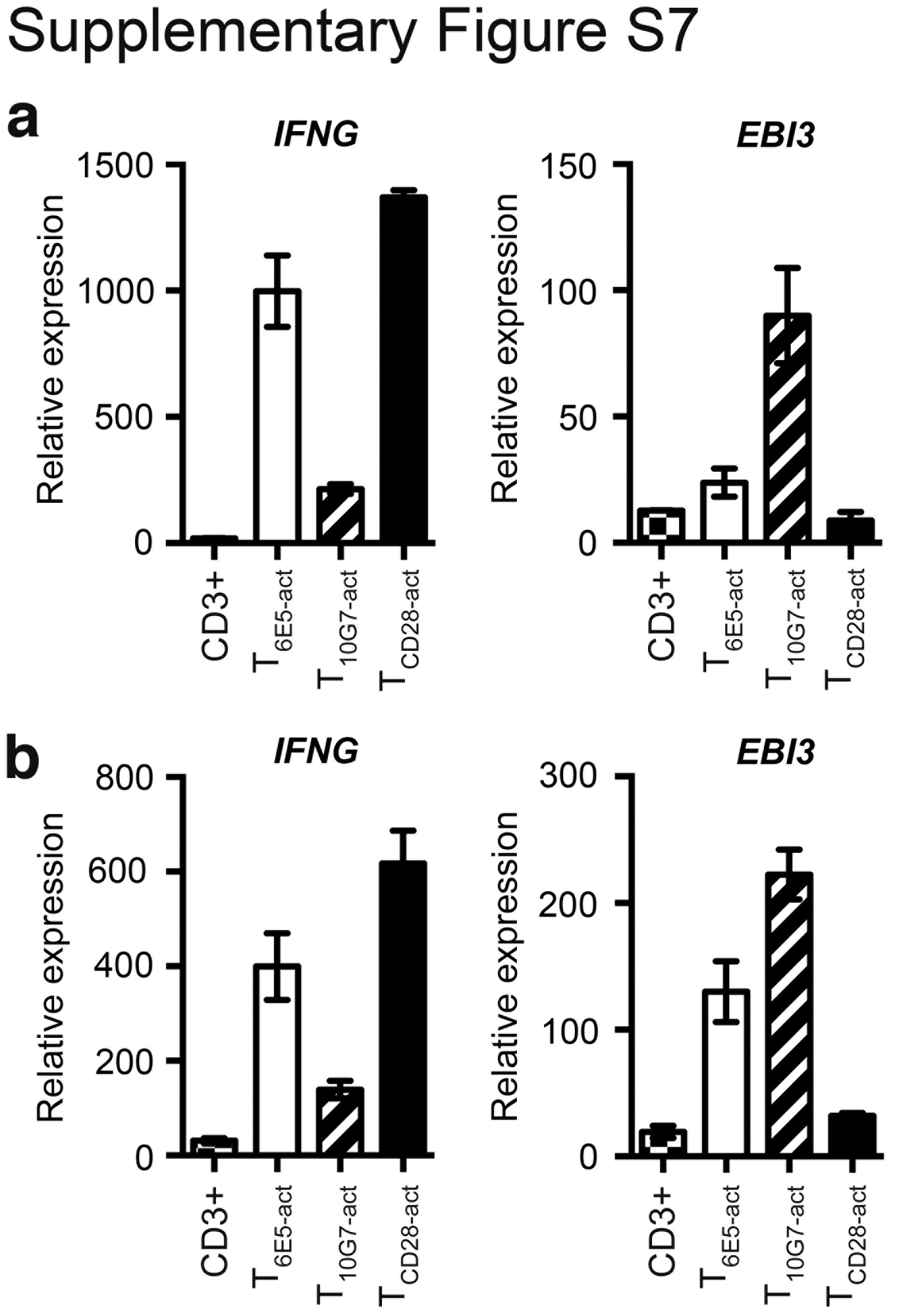

Supplement: Supplementary file 7 — Figure S7. The downstream effect of CD43 co‐stimulation is similar in CD4+ and CD8+ T cells. [file IMM-149-280-s007.tif]
