# Supplementary material for: Engagement of distinct epitopes on CD43 induces different co‐stimulatory pathways in human T cells
Source: Immunology. 2016 Aug 16;149(3):280–96. doi: 10.1111/imm.12642 (PMC5046061; doi:10.1111/imm.12642)
Supplement: Supplementary file 8 — Table S1. Real time PCR primer sequences. [file IMM-149-280-s008.docx]

Supplementary Table S1

Real time PCR primer sequences

| Gene | NCBI ID | Protein ID | Forward Sequence | Reverse Sequence |
| --- | --- | --- | --- | --- |
| *IFNG* | 3458 | IFN-γ | TTCAGCTCTGCATCGTTTTG | TCTTTTGGATGCTCTGGTCA |
| *IL4* | 3565 | IL-4 | GCCACCATGAGAAGGACACT | ACTCTGGTTGGCTTCCTTCA |
| *IL22* | 50616 | IL-22 | TGAGTGAGCGCTGCTATCTG | TGTGCTTAGCCTGTTGCTGA |
| *EBI3* | 10148 | EBI3 | CCTCACAGACTACGGGGAAC | AGTCGGTCATCTGAGGTT GC |
| *p35* | 3592 | IL-12p35 | CCAGAAGGCCAGACAAACTC | GCACAGGGCCATCATAAAAG |
| *p28* | 246778 | IL-27p28 | GCGGAATCTCACCTGCCA | GGAAACATCAGGGAGCTGCTC |
| *FOXP3* | 50943 | Foxp3 | GAAACAGCACATTCCCAGAGTTC | ATGGCCCAGCGGATGAG |
| *CD3E* | 916 | CD3ε | TGAGGGCAAGAGTGTGTGAG | TCCTTGTTTTGTCCCCTTTG |
