# Supplementary material for: Engagement of distinct epitopes on CD43 induces different co‐stimulatory pathways in human T cells
Source: Immunology. 2016 Aug 16;149(3):280–96. doi: 10.1111/imm.12642 (PMC5046061; doi:10.1111/imm.12642)
Supplement: Supplementary file 9 — Table S2. Cytokine profile of T6E5‐act, T10G7‐act and TCD28‐act. [file IMM-149-280-s009.docx]

Supplementary Table S2

Cytokine profile of _T6E5-act_, T_10G7-act_ and T_CD28-act_

|  | Unstimulated | CD3+ | T_6E5-act_ | T_10G7-act_ | T_CD28-act_ |
| --- | --- | --- | --- | --- | --- |
| IFN-γ | 0.84 ± 0.38 | 6.52 ± 0.75 | 18.51 ± 1.25 | 13.21 ± 0.97 | 21.6 ± 1.83 |
| IL-4 | 0.25 ± 0.040 | 7.63 ± 2.63 | 34.04 ± 4.9 | 21.87 ± 5.13 | 40.71 ± 6.29 |
| IL-22 | 0.75 ± 0.14 | 9.12 ± 6.88 | 29.54 ± 5.46 | 20.88 ± 5.12 | 25.62 ± 4.38 |

PB T cells activated via plate bound CD3, CD3/CD43-6E5, CD3/CD43-10G7 and CD3/CD28 for 48 hr. Expression of IFN-γ, IL-4 and IL-22 was analyzed by intracellular cytoplasmic staining. Data show percentage of positive cells (no. of experiments= 2, no. of donors= 2). Data show mean ± SEM.
