# Supplementary material for: Engagement of distinct epitopes on CD43 induces different co‐stimulatory pathways in human T cells
Source: Immunology. 2016 Aug 16;149(3):280–96. doi: 10.1111/imm.12642 (PMC5046061; doi:10.1111/imm.12642)
Supplement: Supplementary file 10 [file IMM-149-280-s010.docx]

**Supplementary Figure S1**

Reactivity profile of CD43 mAbs CD43-6E5 and CD43-10G7 on human T cells

(a) Co-expression of CD43-6E5 (FITC) and CD43-10G7 (PE) on unstimulated PB T (ii) and on PB T cells stimulated with PMA/ionomycin (100 nM) for 12 hr (iv). Reactivity of isotype control is also indicated (i, iii). Gate was set on live T cell population (not shown). Quadrant marker was set according to results obtained with isotype control. Data are representative of two independent experiments with four different donors.
(b) Expression profile of CD43 mAbs 6E5 (top panel), 10G7 (bottom panel) on PB CD4^+^ T (grey filled histograms) and PB CD8^+^ T cells (thick open histograms). Reactivity of isotype control is also indicated (dotted open histograms). Gate was set on live T cell population (not shown). Data are representative of two independent experiments with two different donors for CD4^+^ as well as CD8^+^ T cells. (c) Binding profile of CD43 mAb 6E5 (🞆) and 10G7 (◆) on PB T cells. Data are representative of three independent experiments with three different donors. (d) Data show fold change in the expression of CD43-6E5 () and CD43-10G7 (◼) on PB T cells analyzed at 4°C (dotted line) and at 37°C (thick line) at indicated time points. Data are representative of two independent experiments with two different donors. (e) Reactivity of CD43-6E5 (top panel) and of CD43-10G7 (bottom panel) on Bw-CD43 cells (thick open histograms); on Bw5417 control cells (grey filled histograms). Reactivity of isotype control antibody is also shown (thin open histograms). Gate was set on live cell population (not shown). Data are representative of three independent experiments. (f) Reactivity of CD43-6E5 (grey filled histogram) and of CD43-10G7 (thick open histogram) on Jurkat E6 cells. Reactivity of isotype control antibody is also shown (thin open histograms). Gate was set on live cell population (not shown). Data are representative of three independent experiments.

**Supplementary Figure S2**

Down-modulation of CD43 surface expression by CD43-6E5 requires TCR signaling

(a and b) Expression profile of CD43-6E5 (a) and CD43-10G7 (b) (thick filled histograms) on unstimulated T cells, T cells stimulated via plate bound CD43-6E5 for indicated time points. Reactivity of isotype control is also shown (dotted filled histograms). Numbers indicate percentage of positive cells. Gate was set on live cell population (not shown). Data shown are representative of two independent experiments with two different donors.

**Supplementary Figure S3**

CD4^+^, CD8^+^ PB T and CB T cell stimulation upon engagement of CD43 mAbs

(a) PB T cells were activated via plate bound CD43 mAbs or CD28 mAb without CD3 stimulation. T cell proliferation was measured by analyzing [methyl-^3^H]-thymidine incorporation at day 3. (b) CB T cells were activated via plate bound CD43 mAbs or CD28 mAb along with CD3 mAb. T cell proliferation was measured by analyzing [methyl-^3^H]-thymidine incorporation at day 3 (no. of experiment= 5, no. of donors= 5). Data show mean ± SEM. (c) PB CD4^+^ (i) and CD8^+^ (ii) T cells were activated via plate bound CD43 mAbs or CD28 mAb along with CD3 mAb. T cell proliferation was measured by analyzing [methyl-^3^H]-thymidine incorporation at day 3. (a and c) Data are representative of two independent experiments with two different donors. Data show mean ± SD. (d) PB T cells were activated with respective plate-bound mAbs and analyzed after 48 hr for the expression of various cell surface markers by flow cytometry. Gate was set on live T cell population (not shown). Numbers indicate percentage of positive cells. Data show expression profile of indicated mAbs on unstimulated T cells, T_6E5-act_, T_10G7-act_ and T_CD28-act_. Data shown are representative of three independent experiments with three different donors. (e) Images of unstimulated PB T cells, PB T cells activated via immobilized mAbs CD43-6E5, CD43-10G7, CD3, CD3/CD43-6E5, CD3/CD43-10G7 and CD3/CD28. Data are representative of five independent experiments with five different donors.

**Supplementary Figure 4**

T_10G7-act_ acquire FOXP3 independent suppressive function

(a) Bar diagram represents percentage of suppression exerted by TReg, T_6E5-act_, T_10G7-act_ and T_CD28-act_ when used at ratio 1:1 with effector T cells in allogeneic MLR. Data show mean ± SEM (* p<0.05, ** p<0.01, *** p<0.001) (no. of experiments= 3, no. of donors= 3) (b) Expression of FOXP3 analyzed by intracellular staining, in PB T cells activated via respective mAbs for 48 hr. The cells were first stained for cell surface expression of CD45RA. The cells were then fixed and stained intracellularly for FOXP3 and Isotype control. Numbers indicate percentage of positive cells within viable cell population. Data shown are representative of two independent experiments with two different donors.

**Supplementary Figure S5**

Heterotypic interaction of DC with pre-activated T cells is an active process

(a) Images of irradiated T cells activated via immobilized mAbs CD3/CD43-6E5, CD3/CD43-10G7 and CD3/CD28. (b) Images of irradiated DC co-cultured with irradiated T_6E5-act_, T_10G7-act_ and T_CD28-act_ (iii). (a-b) Data are representative of two independent experiments.

**Supplementary Figure S6**

Gating strategy for flow cytometry analysis of cluster formation between DC and T cells

(a) Quadrant markers were set according to results obtained from CellTrace^TM^ Oregon Green 488® stained DC and CellTracker^TM^ Red CMPTX stained T_6E5-act_, T_10G7-act_ and T_CD28-act_. Numbers in the quadrant indicate percentage of positive cells. (b) Based on forward and side scatter the whole cell population was divided into three subpopulations. Numbers within the gate R1, R2 and R3 indicate percentage of cells from total cell number. Numbers in the quadrant represent the percentage of cells within the respective gates (R1/R2/R3). Double positive cells were mostly found in R1. Therefore for further analysis the R1 cell population was considered.

**Supplementary Figure S7**

The downstream effect of CD43 co-stimulation is similar in CD4^+^ and CD8^+^ T cell

(a-b) Expression of *EBI3* and *IFNG* by qPCR in PB CD4^+^ T cell (a) and CD8^+^ T cells (b) activated via respective mAbs for 30 hr. Data show ± SD. Data are representative of two independent experiments with two different donors for CD4^+^ as well as CD8^+^ T cells.
